# Real-Time Tunable Gas Sensing Platform Based on SnO_2_ Nanoparticles Activated by Blue Micro-Light-Emitting Diodes

**DOI:** 10.1007/s40820-024-01486-2

**Published:** 2024-08-08

**Authors:** Gi Baek Nam, Jung-El Ryu, Tae Hoon Eom, Seung Ju Kim, Jun Min Suh, Seungmin Lee, Sungkyun Choi, Cheon Woo Moon, Seon Ju Park, Soo Min Lee, Byungsoo Kim, Sung Hyuk Park, Jin Wook Yang, Sangjin Min, Sohyeon Park, Sung Hwan Cho, Hyuk Jin Kim, Sang Eon Jun, Tae Hyung Lee, Yeong Jae Kim, Jae Young Kim, Young Joon Hong, Jong-In Shim, Hyung-Gi Byun, Yongjo Park, Inkyu Park, Sang-Wan Ryu, Ho Won Jang

**Affiliations:** 1 Department of Materials Science and Engineering, Seoul National University, Seoul, 08826 Republic of Korea; 2 Research Laboratory of Electronics, Department of Mechanical Engineering, Massachusetts Institute of Technology, Cambridge, MA 02139 USA; 3 Ming Hsieh Department of Electrical and Computer Engineering, University of Southern California, Los Angeles, CA 90089 USA; 4 Department of Display Materials Engineering, Soonchunhyang University, Asan, 31538 Republic of Korea; 5 Department of Photonics and Nanoelectronics, BK21 FOUR ERICA-ACE Center, Hanyang University ERICA, Ansan, 15588 Republic of Korea; 6 Department of Nanotechnology and Advanced Materials Engineering, Sejong University, Seoul, 05006 Republic of Korea; 7 Department of Electronics, Information and Communication Engineering, Kangwon National University, Samcheok, 25913 Republic of Korea; 8 Advance Institute of Convergence Technology, Seoul National University, Suwon, 16229 Republic of Korea; 9 Department of Mechanical Engineering, Korea Advanced Institute of Science and Technology, Daejeon, 34141 Republic of Korea; 10 Department of Physics, Chonnam National University, Gwangju, 500-757 Republic of Korea

**Keywords:** Micro-LED, Gas sensor array, Low power consumption, Metal decoration, Real-time detection

## Abstract

Micro-light-emitting diodes (μLEDs) have gained significant interest as an activation source for gas sensors owing to their advantages, including room temperature operation and low power consumption. However, despite these benefits, challenges still exist such as a limited range of detectable gases and slow response. In this study, we present a blue μLED-integrated light-activated gas sensor array based on SnO_2_ nanoparticles (NPs) that exhibit excellent sensitivity, tunable selectivity, and rapid detection with micro-watt level power consumption. The optimal power for μLED is observed at the highest gas response, supported by finite-difference time-domain simulation. Additionally, we first report the visible light-activated selective detection of reducing gases using noble metal-decorated SnO_2_ NPs. The noble metals induce catalytic interaction with reducing gases, clearly distinguishing NH_3_, H_2_, and C_2_H_5_OH. Real-time gas monitoring based on a fully hardware-implemented light-activated sensing array was demonstrated, opening up new avenues for advancements in light-activated electronic nose technologies.

**Supplementary Information:**

The online version contains supplementary material available at 10.1007/s40820-024-01486-2.

## Highlights

- Blue micro-light-emitting diodes (μLED)-integrated gas sensors were fabricated as monolithic structure by directly loading sensing materials onto the μLED.
- SnO_2_ nanoparticles are activated by blue μLED and exhibit outstanding sensitivity to NO_2_ at *μ*-Watt power levels.
- Noble metal (Au, Pd, Pt)-decorated SnO_2_ showed the tunable gas selectivity for 4 target gases under blue light illumination.

**Supplementary Information:**

The online version contains supplementary material available at 10.1007/s40820-024-01486-2.

## Graphical abstract

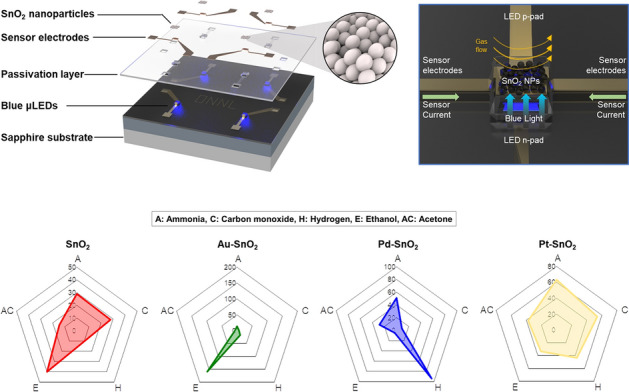

## Introduction

The gradual increase of the pandemic has enlightened the public about the significance of real-time detection of hazardous substances in daily life. Specifically, the detection of gaseous analytes has become more essential due to the direct association with human health. The growing demand for harmful gas detection has triggered the development of various types of gas sensors, including electrochemical sensors, surface acoustic wave sensors, optical sensors, and chemoresistive sensors [1, 2]. Among these, chemoresistive gas sensors have gained tremendous attention owing to their simple structure, small size, and facile operation. The fundamental operation principle is based on measurement of the change in electrical current of the sensing materials upon exposure to the target gas. Metal oxides are the most widely utilized materials in chemoresistive gas sensors [3]. However, at room temperature, metal oxides-based chemoresistive sensors suffer from low response and irreversible recovery [4]. To overcome these drawbacks, external heating systems were adopted to elevate the operation temperature, including back heater and microelectromechanical (MEMS) systems [5, 6]. Despite improved gas sensing properties through thermal activation, high power consumption and thermal degradation of sensing materials remain critical issues, which are unfavorable for miniaturization and stability. Thus, extensive research on room temperature operable gas sensors for high reliability and stability has been extensively conducted.

Recently, light activation using light-emitting diode (LED) has been extensively utilized for room temperature operation with reduced power consumption [7, 8]. Light irradiation on the sensing material generates the photo-carriers and forms the reactive photo-activated oxygen, which is reactive with target gases [9, 10]. Ultraviolet (UV) light has been widely utilized in the activation of metal oxides due to the large bandgap of metal oxides [11]. However, UV light is known to harm the human body and cause skin-related diseases such as skin cancer, premature aging, and burns [12, 13]. To address this drawback, in our previous work, we fabricated SnO_2_ nanoparticles (NPs) gas sensor activated by visible light illumination, which does not harm human skin [14]. The defect states in SnO_2_ NPs enabled the photoexcitation under visible light, resulting in room temperature NO_2_ detection with high response and selectivity. Despite these promising features, light-activated gas sensors based on SnO_2_ NPs still have limitations that need to be addressed. The use of commercial LEDs requires high power consumption compared to MEMS heaters, and the distance between the LED and the sensing material results in energy loss due to light diffusion. Furthermore, most research on light-activated gas sensors has focused on a single target gas detection, especially NO_2_. Detecting a variety of hazardous gases, such as NO_2_, NH_3_, H_2_, and VOCs, is essential for protecting human health. However, since NH_3_, H_2_, and VOCs are less reactive than NO_2_ at room temperature, it is challenging to detect these gases using visible light-activated techniques at room temperature [4, 15].

Herein, we introduce a fully hardware-implemented blue μLED-integrated gas sensor array based on noble metal decorated SnO_2_ NPs with controllable gas selectivity. By using μLED instead of commercial LED, power consumption is significantly reduced from milli-watt to micro-watt. Furthermore, the μLED-integrated gas sensor reduces the distance between the sensing material and the light source, which can substantially be efficient to power consumption and increase energy efficiency. The compact configuration allows for application on various platforms, including wearable devices, medical devices, and smart home systems, expanding the potential applications of μLED-integrated gas sensors. SnO_2_ NPs, previously studied as sensing materials, were deposited on μLED-integrated gas sensor [14]. Under the blue light, the 10 nm-sized SnO_2_ NPs exhibited excellent sensing performance, including high response, fast response and recovery, high reliability, and low detection limit. Finite-difference time-domain (FDTD) simulation was conducted to reveal the mechanism of the characteristics of gas response. Furthermore, noble metals (Au, Pd, and Pt) were decorated on SnO_2_ NPs to detect gases such as NH_3_, H_2_, and C_2_H_5_OH with fabrication of μLED gas sensor array. While several studies have been published that apply noble metal decoration to modulate gas selectivity, these studies are limited to high temperature environments [16, 17]. By applying noble metal decoration on SnO_2_ NPs, which has superior light activation properties, the catalytic effect of noble metals has enabled selective gas detection under blue light illumination. Moreover, the real-time wireless gas monitoring was demonstrated with fully μLED-hardware-integrated gas sensor array by utilizing a microcontroller unit (MCU) and customized printed circuit board (PCB). This study will establish a guideline and pave the new avenue for advancement in electronic nose (e-nose) technologies.

## Experimental Section

### Fabrication of μLED Platform

μLED gas sensor platform was fabricated by following procedures. Conventional LED on sapphire wafer with 8 pairs of superlattices and 4 pairs of multiple quantum wells was prepared into chip scale. A 2 × 2 array of square patterns was formed on the LED film via photolithography followed by inductively coupled plasma etching to vertically etch the LED film to form a mesa structure. Then, the current spreading layer composed of 10 nm Ni and 10 nm Au was deposited on the p-type GaN region by photolithography and e-beam evaporation. After the lift-off process of Ni/Au metal stack, the sample was annealed at 500 °C in ambient air by a rapid thermal annealing (RTA) system to form an ohmic contact between the p-type GaN layer and current spreading layer by oxidizing the Ni layer [18–20]. The ultra-thin Ni/Au layer would turn into a transparent layer after the thermal annealing process. After that, n-type contact metal, composed of 20 nm Cr for adhesion and 200 nm Au for current flow, was formed on the n-type GaN layer by photolithography and e-beam evaporation. To connect the current spreading layer to the p-type contact metal that would be formed later, a small region of the Cr/Au metal layer was also formed on the current spreading layer during the process. For electrical insulation, 700 nm SiO_2_ was deposited on the sample by plasma-enhanced chemical vapor deposition (PECVD). The contact region of the metal layer was opened by photolithography and reactive ion etching (RIE). On top of that, the lift-off pattern was formed by photolithography, and p-type contact metal also composed of 20 nm Cr and 200 nm Au was deposited by e-beam evaporation. An additional insulation layer of 700 nm thick SiO_2_ was deposited by PECVD followed by RIE etching to expose the contact region. Finally, 20 nm Cr and 200 nm Au electrodes were deposited on the LED via photolithography and e-beam evaporation. The overall fabrication process of μLED platform is presented in Fig. S1.

### Preparation of SnO_2_ NPs, Au-SnO_2_ NPs, Pd-SnO_2_ NPs, and Pt-SnO_2_ NPs

SnO_2_ NPs were prepared by following the procedures of our previous work [14]. 3 g of SnCl_4_·5H_2_O was dissolved in 60 mL of deionized (DI) water and stirred for 1 h. The dissolved solution was transferred to a Teflon-lined autoclave for hydrothermal synthesis. The synthesis was conducted in a dry oven at 200 °C for 24 h. After sufficient cooling to room temperature, the synthesized products were collected after the cleaning process by centrifugation. The centrifugation was conducted at 10,000 rpm for 10 min, which was repeated 3 times. The powders were dried on a hot plate for 12 h to remove the solutions. For noble metal decoration, SnO_2_ NPs were dispersed in DI water (1 mg mL^−1^) and ultrasonicated for 1 h. The metal precursors (HAuCl_4_, K_2_PdCl_4_, and K_2_PtCl_4_) were dissolved in DI water (1 mg mL^−1^) and ultrasonicated for 30 min. For Au decoration, 0.2 mg of HAuCl_4_ and 0.2 mg of Pluronic F-127 were added to 1 mg of SnO_2_ dispersion under ultrasonication for 10 min. For Pd decoration, 0.2 mg of K_2_PdCl_4_ and 0.2 mg of Pluronic F-127 were added to 1 mg of SnO_2_ dispersion under constant stirring for 1 h. For Pt decoration, 0.2 mg of K_2_PtCl_4_ and 0.2 mg of Pluronic F-127 were added to 1 mg of SnO_2_ dispersion under constant stirring for 3 h at 100 °C. The centrifugation was conducted to remove the residues after the decoration process and doping process. The products were collected after completely drying the powders.

### Gas Sensing Measurements

For gas sensing measurements, a slurry was prepared by mixing SnO_2_ NPs with a terpineol-based ink. The ink was then uniformly coated on Cr/Au electrodes and dried on a hot plate. This process was repeated three times to fully cover the sensing area of Cr/Au electrodes. The RTA was conducted under N_2_ atmosphere at 350 °C for 10 min to improve the crystallinity of SnO_2_ NPs and evaporate the solvent. The prepared sensor was placed inside a quartz tube, and voltage was applied to both μLED electrodes and sensing electrodes. The resistance was recorded by *I-V* source meter (Keithley 2625) under an applied voltage of 0.5 V. Dry air was continuously supplied inside the quartz tube with the assistance of mass flow controllers (MFC). The target gases, prepared in air balance, were inserted into the quartz tube at specific concentrations. The gas concentration was controlled by adjusting the flow ratio of air and the target gases, maintaining a total flow rate of 1000 sccm. Humid air was generated by passing dry air through a water bubbler. To control the humidity level, the generated humid air was mixed with additional dry air using MFC. Real-time gas monitoring of the μLED gas sensor array was conducted in a desiccator to control gas environment.

### Characterizations

The electrical properties of the fabricated μLEDs were characterized by Keithley 4200A. For optical measurement, a source meter (Keithley 2602B) was used for supplying the current to the sample, while a Si p-i-n photodiode (Hamamatsu Photonics S2281–04) and a fiber-optic spectrometer (AvaSpec-2048) were utilized for analysis. The morphology of SnO_2_ NPs was characterized by field emission scanning electron microscope (FE-SEM, Hitachi, SU70) and transmission electron microscope (TEM, JEM 2100F). X-ray diffraction (XRD) analysis was conducted by D8-advance, BRUKER MILLER Co. X-ray photoelectron spectroscopy (XPS) spectra were obtained by AXIS-HIS, KRATOS with Al Kα (1486.6 eV) X-ray source at 25.3 W. The UV–Vis absorbance spectra were measured by UV–Vis spectroscopy (770, JASCO), and photoluminescence (PL) spectrum was recorded by a micro-PL system (Dongwoo Optron) with a 325 nm He-Cd laser. The laser spot size of the micro-PL system used in this study was 3 µm. The investigation on substrate temperature change under LED illumination was conducted by IR camera (AX5 series, FLIR).

### Finite-Difference Time-Domain Simulation

Electromagnetic simulations proceed with a finite-difference time-domain (FDTD) program (Lumerical Solutions). Refractive indices of SnO_2_ and SiO_2_ are adopted from Mohamed [21] and Palik [22]. 36 layers of 10-nm-sized SnO_2_ NPs with close-packed FCC structure are arranged in over SiO_2_ planar thin film in air. Periodic boundary conditions were adopted for the x and y dimensions, and perfectly matched layer conditions were adopted for the z dimension. A plane-wave source is adopted, and it propagates from − z to + z (forward) direction (SiO_2_ to air). An unpolarized wave is adopted by averaging s and p-polarized waves. A 0.5-nm-sized cubic size cell was used for meshing.

## Results

### Characterization of μLED-Integrated Gas Sensor Array

**Fig. 1:**
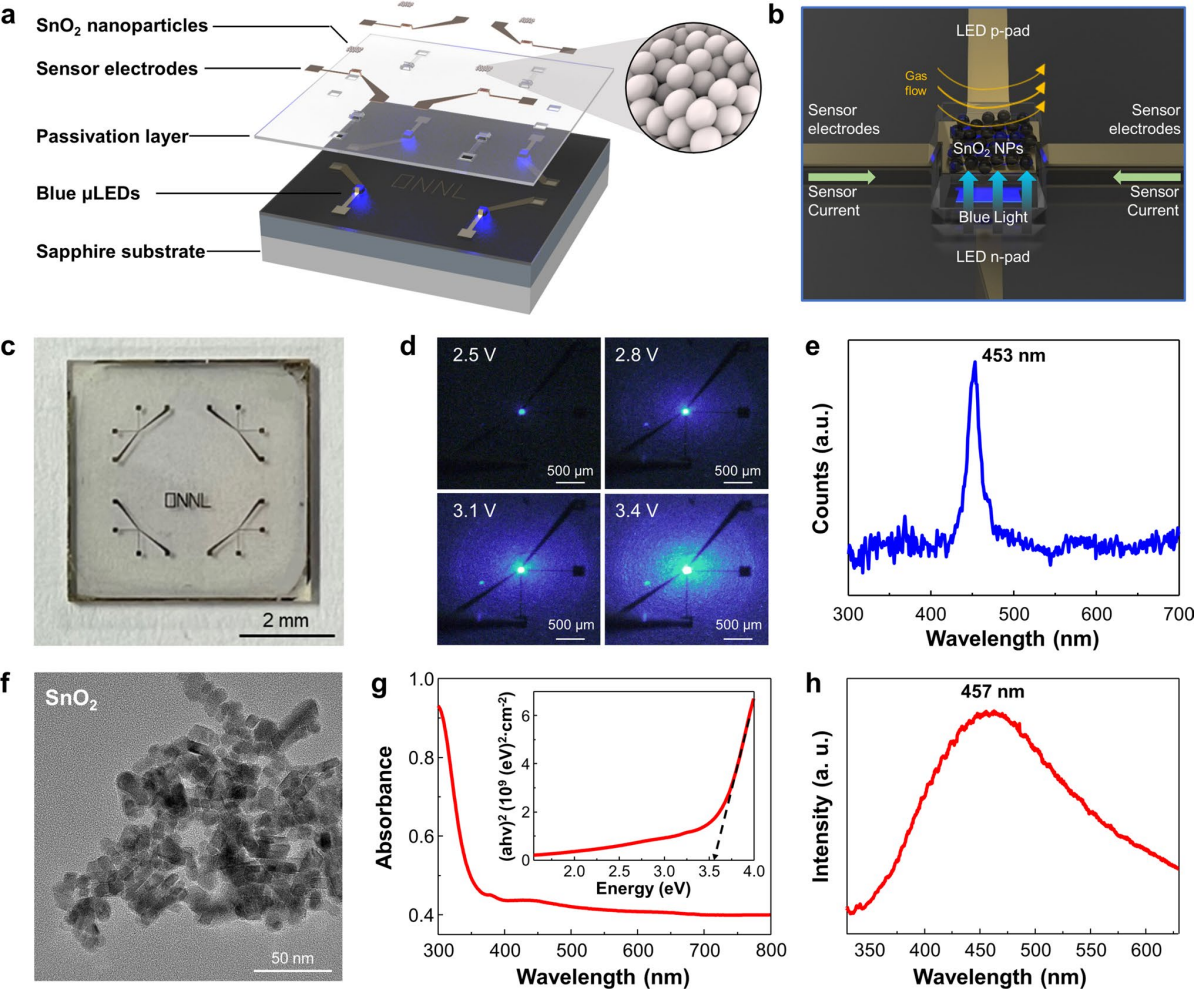
µLED-integrated gas sensor array with SnO_2_ NPs. Schematic illustration of **a** structure, and **b** process to gas detection of µLEDs-integrated gas sensor. **c** Optical image of µLEDs-integrated gas sensor array. **d** Optical images of µLEDs-integrated gas sensor array with different light intensities. **e** PL spectrum of µLEDs. **f** TEM image, **g** absorbance plot and Tauc plot, and **h** PL spectrum of SnO_2_ NPs

Figure 1a presents the simplified structure of the μLED platform for gas sensing measurement. The device consists of 4 individual μLEDs to fabricate gas sensor array for discrimination of different target gases. The optical image of μLED gas sensor platform with dimensions of 20 × 20 μm^2^ is shown in Fig. S2. Each μLED has p- and n-contact electrodes for operation and sensor electrodes for gas detection with a SiO_2_ passivation layer between them. The light passes through the SiO_2_ layer and photo-activates the sensing material on the device, which is located between the two sensing electrodes, as shown in Fig. S3 [23, 24]. Figure S4 shows the transmittance of SiO_2_ layer, indicating that the SiO_2_ passivation layer has little effect on the light emitted by the blue μLED. Figure 1b illustrates the enlarged structure image, wherein SnO_2_ NPs were directly loaded onto the μLEDs platform, establishing electrical connections with the sensor electrodes. The blue light emitted from the μLEDs activates SnO_2_ NPs, while the target gas is introduced to the device through a quartz tube and reacts with SnO_2_ NPs. The resistance signal is read from the sensor electrodes as target gases react with SnO_2_ NPs. Figure 1c shows a photograph of a 2 × 2 array of μLED-integrated gas sensor platform, with the device of 1 × 1 cm^2^. The sensing material was coated using a micro-needle, as shown in Fig. S5a. The ink droplet was prepared by dispersing the sensing material in the terpineol-based solvent. The microneedle was dipped into the prepared ink, forming a small droplet. The ink droplet was cast on the sensing area, as displayed in the optical microscope image in Fig. S5b. The sensing material covered the LED region and successfully filled the space between the sensor electrodes, electrically connecting the two electrodes. The uniform coating of the sensing material on the μLED was confirmed by SEM image in Fig. S6. Using this method, the sensing materials are deposited in a cost-effective and simple manner without the need for additional lithography steps. Figure 1d shows emission images of the μLED under different applied voltages: 2.8, 3.1, 3.4, and 3.7 V. The electrical properties of μLED are presented in Fig. S7a, where ohmic contact formation was confirmed from the current–voltage (*I-V*) measurement, with an estimated turn-on voltage of 3.4 V. The effect of the SiO_2_ insulating layer was confirmed by the extremely low reverse leakage current of 1.2 × 10^–10^ A at − 5 V as presented in the inset of Fig. S7a. Figure S7b depicts the emission intensity of μLED under different injection currents, where the emission intensity demonstrates an apparent linear increase, supporting the formation of ohmic contact at the interface between the current spreading layer and p-type GaN. The device emitted a sharp blue light characterized by a peak wavelength of 453 nm and a full width at half maximum (FWHM) value of 20.95 nm at an operating power of 63.2 µW, as shown in Fig. 1e. External quantum efficiency (EQE) of the 20 × 20 μm^2^ sized μLED was measured to be 2.91%, as shown in Fig. S8. The EQE of μLED was measured to be lower than the EQE of commercial LEDs due to the size effect of the μLEDs [25–27]. However, the low EQE has a marginal effect on the sensing performance, as the sensing material is located directly above the μLED. The distance between the light source and sensing material is less than 1.5 μm, minimizing the energy loss of light emitted from the μLED. As depicted in Fig. S9, the μLED exhibited a blue shift of the emission wavelength as the injection current increased due to the quantum confined Stark effect (QCSE) [28, 29]. However, our device operates at a low current level, minimizing the effect of QCSE on emission performance. Overall, the μLED platform was successfully fabricated and emitted sharp blue light with extremely low power consumption.

For gas sensing material, SnO_2_ NPs were prepared by hydrothermal method. SnO_2_ is a direct bandgap material, which can effectively absorb photon emitted from μLED [30]. Furthermore, SnO_2_ has dual valency with an Sn oxidation states of Sn^2+^ and Sn^4+^ [31]. This dual valency of Sn facilitates the formation of Sn interstitials and oxygen vacancies with low formation energy [32]. These interstitial and vacancy sites generate the trap sites between conduction band and valance band, enabling SnO_2_ to absorb light in visible range, which is lower energy than the bandgap of SnO_2_ [33]. Figure 1f showed SnO_2_ NPs with an average particle size of 10 nm. High-resolution images were obtained using TEM to investigate the crystallinity of the SnO_2_ as shown in Fig. S10. The crystal structure of each material was confirmed with diffraction patterns, which are presented in the inset image. The lattice fringe was observed to be 0.33 nm, corresponding to the (110) plane of SnO_2_ [34, 35]. The XRD spectra of SnO_2_ NPs exhibited clear peaks, which indicates the formation of rutile SnO_2_ validated by JCPDS No. 41-1445, as exhibited in Fig. S11 [31, 36]. The chemical state of SnO_2_ was studied by XPS analysis, as depicted in Fig. S12. The Sn 3*d*_3/2_ and Sn 3*d*_5/2_ peaks of SnO_2_ appeared at 495.05 and 486.65 eV, respectively. The binding energy difference between Sn 3*d*_3/2_ and Sn 3*d*_5/2_ peak was measured to be 8.4 eV, which is in good agreement with the chemical state of Sn^4+^ [37, 38]. The O 1*s* spectra of SnO_2_ NPs were subjected to deconvolution, resulting in the identification of three distinct peaks corresponding to different oxygen states. The O_ad_ peak, which indicated the oxygen adsorbed on the surface of SnO_2_, appeared at 531.75 eV. The O_V_ peak, representing oxygen vacancy in SnO_2_, appeared at 530.75 eV, while the O_L_ peak, corresponding to oxygen at regular lattice sites of SnO_2_, appeared at 530.25 eV.

UV–vis spectroscopy was employed to evaluate the optical characteristics of SnO_2_, as shown in Fig. 1g. The bandgap was determined by analyzing the absorbance spectrum with a Tauc plot, as shown in the inset image. The bandgap of SnO_2_ was determined to be 3.6 eV, which is a commonly observed value for SnO_2_ [39, 40]. Photoluminescence (PL) analysis of SnO_2_ NPs was conducted, as shown in Fig. 1h. The spectrum exhibited a wide range of energy states, suggesting that the presence of tin interstitials and oxygen vacancies led to the formation of intermediate energy levels [41]. Due to the presence of vacancy states, SnO_2_ NPs could be activated by blue light despite its high bandgap energy. The peak emission wavelength of SnO_2_ NPs was measured to be approximately 450 nm, which is comparable to the emission wavelength of our μLEDs. The optical analyses confirmed that SnO_2_ NPs can be activated within the visible range, particularly by blue light.

### Gas Sensing Performance of SnO_2_ NPs

I-V characteristic curves of SnO_2_ NPs under different light intensities were measured to investigate the photocurrent of SnO_2_ NPs, as shown in Fig. S13. The operating power was calculated using Eq. (1):

1 $$P = I \times V$$

**Fig. 2:**
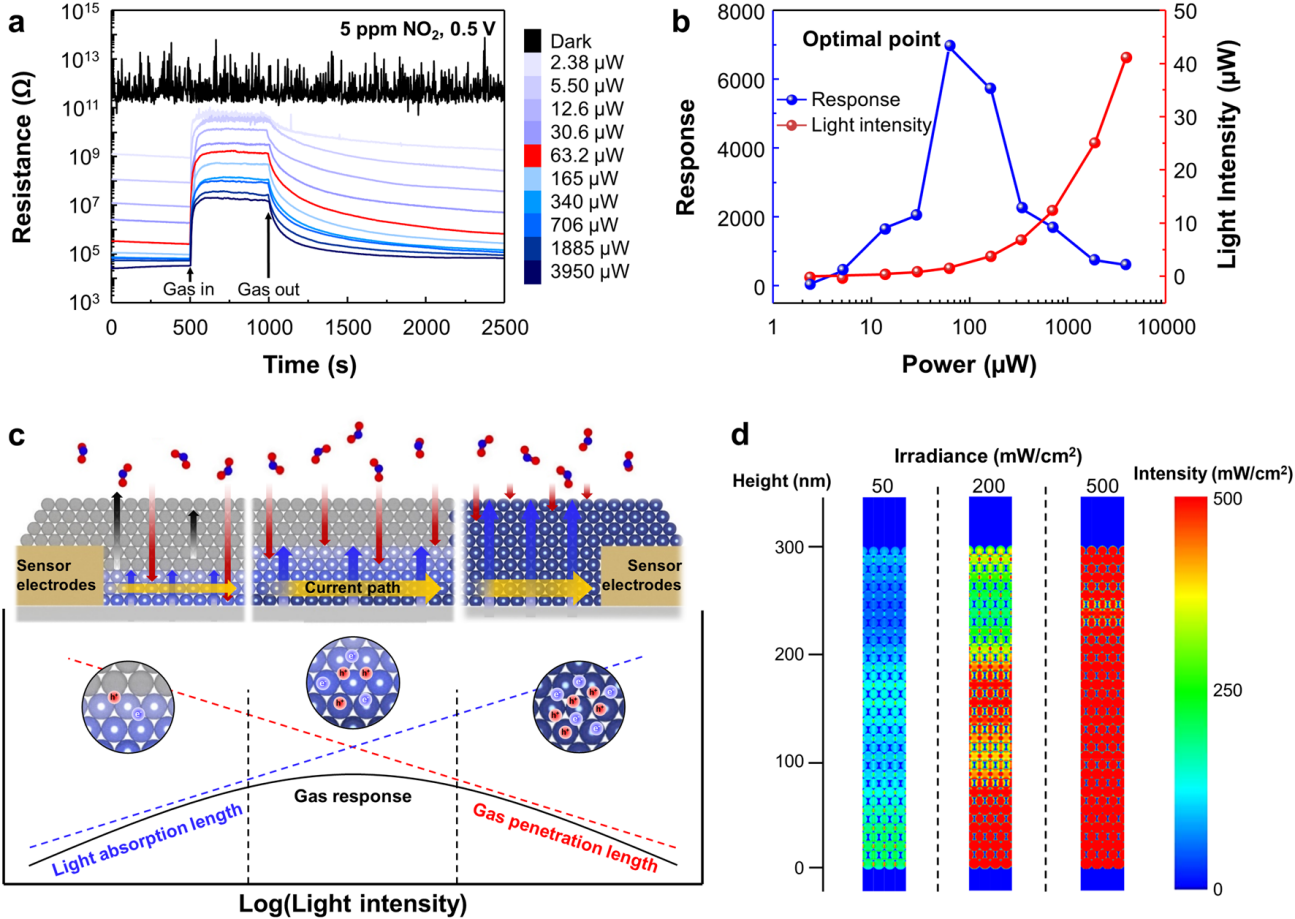
NO_2_ gas responses of SnO_2_ NPs under different light intensities. **a** Dynamic gas sensing curves to 5 ppm of NO_2_ under different light intensities. **b** Optimization of NO_2_ response by injection current. **c** Schematic illustrations of gas sensing reaction under 3 different light intensities. **d** Volume absorption profile of model close-packed SnO_2_ NPs by FDTD simulation. The cross-sectional area is set to 10 × 360 nm^2^

As the power consumption of μLED increased, the photocurrent of SnO_2_ NPs gradually increased. Gas sensing measurements of SnO_2_ were conducted for 5 ppm of NO_2_ under different μLED operating powers, as shown in Fig. 2a. In dark condition, the baseline resistance of SnO_2_ was significantly high, making it difficult to read by sourcemeter, and the response to NO_2_ was discernible. When SnO_2_ is exposed to blue light, photocarriers are generated, which leads to a substantial decrease in the base resistance of SnO_2_. As the power consumption of μLED increased, the baseline resistance decreased, as shown in Fig. S14. When NO_2_ is introduced, SnO_2_ NPs showed the response with an increase in resistance. After 500 s of NO_2_ exposure, air was introduced, and the resistance of SnO_2_ NPs began to recover. The mechanism of light-activation in gas sensors is detailed in Note S1. The relationship between response and power consumption is plotted in Fig. 2b, showing a volcano-shaped plot. The response of NO_2_, denoted as *R*_o_, was defined using Eq. (2), which represents the response to oxidizing gas as the ratio of the resistance variation in the air and target gas atmospheres.

2 $$R_{{\text{o}}} = \, \left( {R_{{\text{g}}} {-}R_{{\text{a}}} } \right)/R_{{\text{a}}}$$

Here, *R*_g_ represents the resistance in the gas atmosphere, while *R*_a_ represents the resistance in the air environment. At low light intensity, the response to NO_2_ increases as the intensity of the light increases. However, the response decreases as the light intensity surpasses the optimal point, which is determined where the response reaches the maximum value. At the optimal point, the response to 5 ppm of NO_2_ was 6928 with a power consumption of 63.2 μW. The response time is defined as the duration required for the resistance to reach 90% of the fluctuation after being exposed to the target gas. As shown in Fig. S15a, the recovery time decreases as the light intensity increases due to the photogenerated holes engaged in NO_2_ desorption. In contrast, the response time increases as the light intensity increases, as shown in Fig. S15b. Higher light intensity increases the number of active sites due to the desorption of adsorbed oxygen, which extends the resistance saturation time. Furthermore, NO_2_ desorption caused by photogenerated holes delays the saturation of NO_2_ adsorption. At the optimal point, the response time and recovery time showed 47 and 49 s. To ensure the effect of light activation, the device temperature was measured both before and after operation, as depicted in Fig. S16. The thermographic images demonstrate no significant temperature variation, indicating that the gas sensing performance is exclusively driven by light activation.

The outstanding response of the μLED gas sensor to NO_2_ can be attributed to both the material characteristics of SnO_2_ and the structural characteristics of the μLED-integrated gas sensor. The detailed material characteristics of SnO_2_ NPs are explained in Note S2. In the case of the structural characteristics, we focused on the geometrical effects of the SnO_2_ NPs on the electrode of the μLED gas sensor to clarify the volcano-like phenomenon in Fig. 2b. Figure 2c illustrates the schematics of the gas sensing reaction categorized into three phases with different light intensities. FDTD simulation was employed to predict the light absorbance of SnO_2_ NPs at different light intensities, as shown in Fig. 2d. SnO_2_ NPs were assumed to possess a spherical shape with densely packed positions. The simulated absorption distribution along the z-axis was obtained for light intensity of 50, 200, and 500 mW cm^−2^, respectively. The first phase in the left panel of Fig. 2c indicates the low light intensity, where the lowest layers are exclusively activated and react with NO_2_. In this phase, all the generated photo-carriers react with the NO_2_ molecules because the number of photo-carriers is considerably smaller than the number of NO_2_ molecules. As the light intensity increases, activation layer of SnO_2_ NPs increases, leading to reactions with more NO_2_ molecules. The response of the sensor reaches maximum value when the activation layer of SnO_2_ NPs is equal to the thickness of sensor electrodes, as shown in the middle panel of Fig. 2c. However, when the activation layers of SnO_2_ NPs become thicker than sensor electrodes due to excessive light intensity, response of the sensor diminishes, as shown in the right panel of Fig. 2c. When the NO_2_ is introduced, most of the NO_2_ molecules react with SnO_2_ NPs located on the top layer. Due to the relatively weak gas reaction in the bottom layers, the resistance of the top layers increases, while the resistance of bottom layers shows little increase. Consequently, the majority of current flows through the bottom layer with low resistance, resulting in a decrease in the response of the sensor. Throughout these three phases, the dependency of the response curve on the light intensity exhibits a volcano-like plot. In conclusion, the material characteristics of SnO_2_ and the structure characteristics of the sensor device generate a synergistic effect on gas sensing performance.

**Fig. 3:**
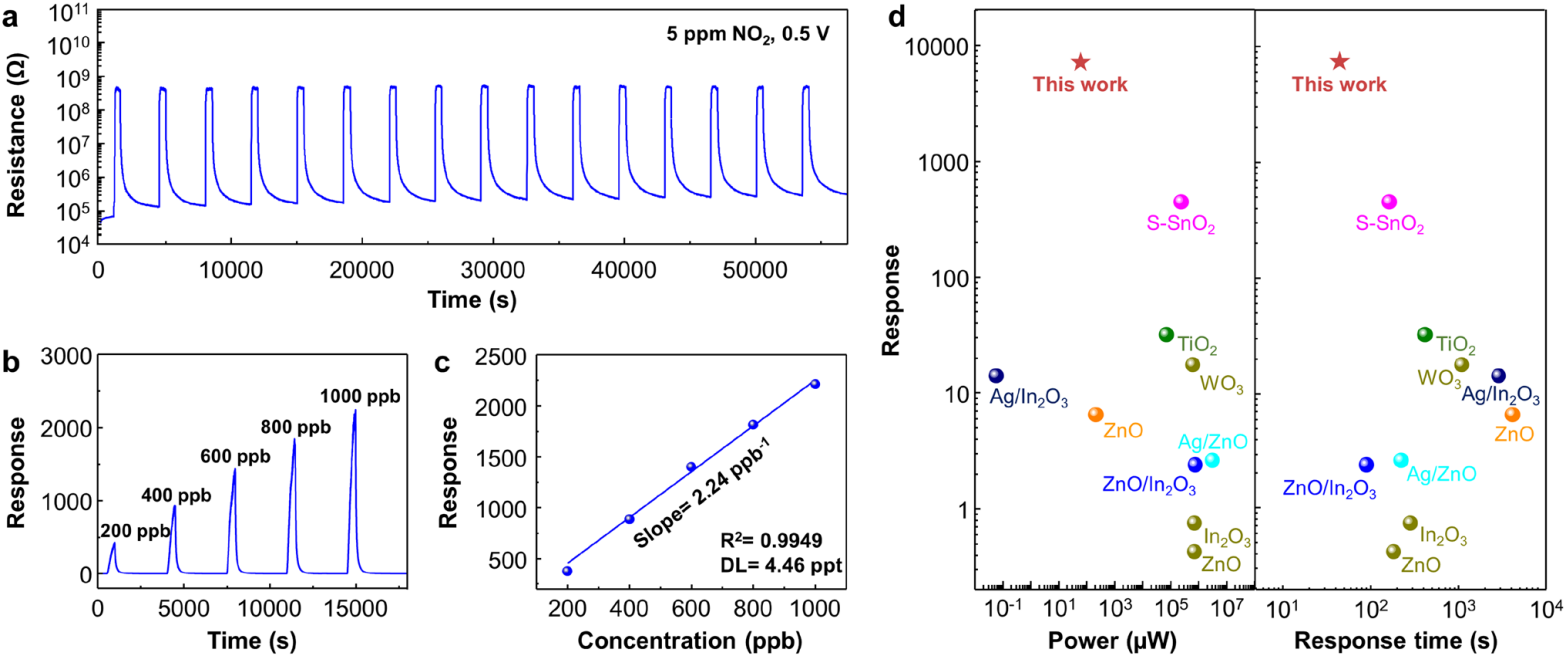
Light-activated NO_2_ sensing properties of SnO_2_ NPs. **a** Response curves to 16 pulses of 5 ppm of NO_2_. **b** Response curves to 200–1000 ppb of NO_2_. **c** Calibration of response and detection limit plot. **d** Comparison of response time vs. response with the state-of-the-art light-activated gas sensors for NO_2_ detection

To evaluate the stability and reliability, the sensor was exposed to 16 repetitive pulses of NO_2_ under blue light illumination with optimal intensity, as shown in Fig. 3a. The sensor demonstrated reliability with sustainable base resistance and response. Figure 3b illustrates the response curves of SnO_2_ to NO_2_ with concentrations ranging from 200 to 1000 ppb. The sensor showed reversible sensing with a high response even at a low gas concentration of 200 ppb. The linear-fitted curve presented in Fig. 3c yielded a slope of 2.24 ppb^−1^ with an *R*^2^ value of 0.9949, supporting the linearity between gas response and NO_2_ concentration. The theoretical detection limit (DL) was determined based on signal-to-noise ratio of 3, using the following equations [9]:

3 $$R_{{{\text{x}}^{2} }} = \sum ((y_{{\text{i}}} - y)^{2}$$

4 $${\text{rms}}_{{{\text{noise}}}} = \sqrt {\frac{{R_{{{\text{x}}^{2} }} }}{N}}$$

5 $${\text{DL}} = 3\frac{{{\text{rms}}_{{{\text{noise}}}} }}{{{\text{slope}}}}$$

where *y*_i_ represents the values of the response curve before NO_2_ exposure, and y denotes the average value of *y*_i_. ${R}_{{\text{x}}^{2}}$ value is calculated by a fifth-order polynomial fitting that extracted 10 data points from *y*_i_. The root means square noise (rms_noise_) is defined as Eq. (5), with *N* representing the number of extracted data points. Through these calculations, the DL was found to be 4.46 ppt under blue light at room temperature. The device uniformity was demonstrated by comparing 3 different sensors, which showed a similar response to 5 ppm of NO_2_, as shown in Fig. S17. The response to 2 ppm of NO_2_ under various humidity states was measured to demonstrate humidity stability, as shown in Fig. S18. As the humidity level increases, the baseline resistance of SnO_2_ NPs decreases. When humid air is introduced to SnO_2_ NPs, H_2_O molecules are adsorbed onto SnO_2_ surface and dissociate into H^+^ and OH^−^ ions, as shown in Eq. (6) [42, 43]. At low humidity levels, the conduction mechanism is proton transport by hopping due to the adsorbed OH^−^ ions [44]. At high humidity levels, the charge transport occurs via the Grotthuss chain reaction, as shown in Eq. (7) [45–47]:

6 $${\text{H}}_{{2}} {\text{O}}_{{({\text{ad}})}} \to {\text{ H}}^{ + } + {\text{ OH}}$$

7 $${\text{H}}_{{2}} {\text{O}}_{{({\text{ad}})}} + {\text{ H}}_{{3}} {\text{O}}^{ + }_{{({\text{ad}})}} \to {\text{ H}}_{{3}} {\text{O}}^{ + }_{{({\text{ad}})}} + {\text{ H}}_{{2}} {\text{O}}_{{({\text{ad}})}}$$

The response to 2 ppm of NO_2_ increased from dry air to RH50% and decreased from RH50% to RH80%. At low humidity levels, pre-chemisorbed H^+^ assists NO_2_ adsorption on SnO_2_, which enhances the response to NO_2_ [48]. In contrast, at high humidity levels, excessive H_2_O adsorbs to Sn active sites in SnO_2_ and fully occupies them with hydroxyl ions (Sn–OH^−^), which inhibits H^+^ enhanced NO_2_ adsorption, leading to a decrease in response to NO_2_. The long-term stability of the sensor to 5 ppm of NO_2_ was investigated by comparing the initial state and after 6 months, as shown in Fig. S19. The sensor maintained a high response and short response time, despite a slight increase in baseline resistance and a decrease in response. The comparison of sensing performance to the state-of-the-art light-activated gas sensors is depicted in Fig. 3d [14, 49–54], and detailed properties are summarized in Table S1. The left panel compares the response to power, while the right panel compares the response to response time. Our device exhibited a response time of 47 s to 5 ppm of NO_2_ with 6928 of response, which is incomparable to other works. Moreover, the response was significantly high with microwatt-scale power consumption (63.2 μW). Furthermore, most reported works utilized UV-LEDs as a light source to activate metal oxide-based gas sensors. In contrast, our device demonstrated superior performance under blue light illumination.

### Noble Metal Decoration on SnO_2_ NPs

**Fig. 4:**
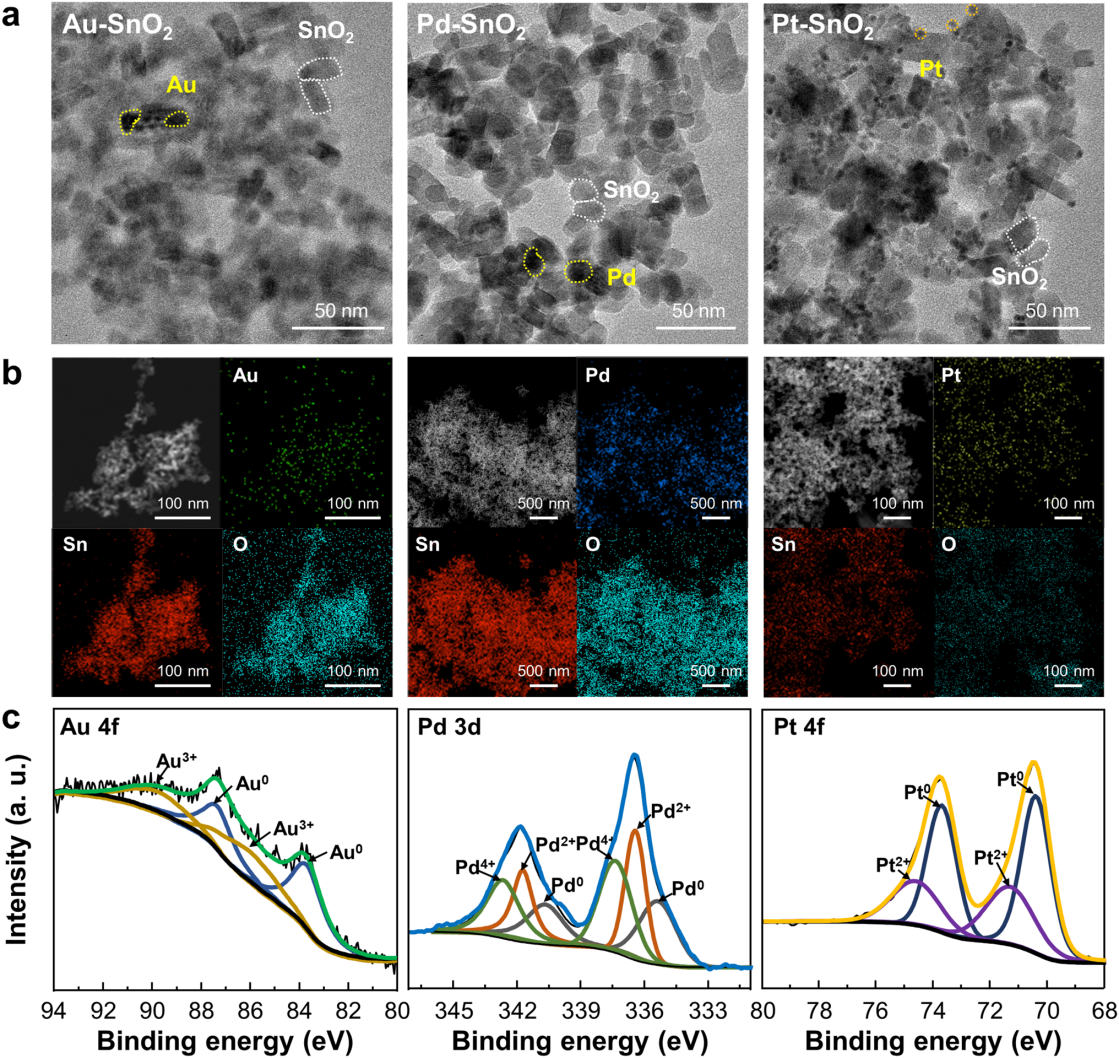
Characterization of noble metal-decorated SnO_2_ NPs. **a** TEM images and **b** EDS element mapping images of Au-SnO_2_ NPs, Pd-SnO_2_ NPs, and Pt-SnO_2_ NPs. **c** Au 4*f*, Pd 3*d*, and Pt 4*f* XPS spectra of Au-SnO_2_ NPs, Pd-SnO_2_ NPs, and Pt-SnO_2_ NPs

The noble metals (Au, Pd, and Pt) were decorated on SnO_2_ NPs to investigate the tunable gas selectivity to reducing gases. For noble metal decoration, SnO_2_ NPs were dissolved in DI water and sonicated to make a colloidal solution. After sonication, noble metal precursors (H_2_AuCl_4_, K_2_PdCl_4_, and K_2_PtCl_4_) were put into SnO_2_ NPs solution. Pluronic F-127 was additionally put into SnO_2_ solution for surfactant and reducing agent [55]. Pluronic F-127 is a block copolymer composed of polyethylene oxide (PEO) and polypropylene oxide (PPO) with a PEO-PPO-PEO triblock structure. Due to the hydrophilic PEO and hydrophobic PPO, Pluronic F-127 constructed a micelle structure in a water-based solution [56]. SnO_2_ NPs were surrounded by Pluronic F-127 and noble metal precursor can permeate into SnO_2_ NPs [57]. Furthermore, Pluronic F-127 facilitated the reduction of metal ions with good stabilization [58]. Overall, through the solution process-based noble metal decoration, Au, Pd, and Pt were uniformly decorated onto the SnO_2_ NPs. Diverse characterizations were conducted to confirm the morphology and chemical states of noble metal NPs. Figure 4a shows TEM images of Au-decorated SnO_2_ NPs (Au-SnO_2_ NPs), Pd-decorated SnO_2_ (Pd-SnO_2_ NPs), and Pt-decorated SnO_2_ (Pt-SnO_2_ NPs). The size of the SnO_2_ NPs was measured to be around 10 nm, whereas Au and Pd were confirmed to be about 5 nm and Pt was measured to be 3 nm. High-resolution images were obtained using TEM to investigate the crystallinity of the Au-SnO_2_ NPs, Pd-SnO_2_ NPs, and Pt-SnO_2_ NPs, as shown in Fig. S20. The crystal structure of each material was confirmed with diffraction patterns, which are presented in the inset of each image. Moreover, STEM with energy-dispersive XPS analysis was performed to observe the distribution of the noble metal NPs on the SnO_2_ NPs. As shown in the EDS images in Fig. 4b, noble metal NPs were well-dispersed throughout each sample. XPS analysis was conducted to confirm the surface chemical states of the noble metal-decorated SnO_2_ NPs. Figure S19 presents the XPS spectra of Sn 3*d* and O 1*s* of Au-SnO_2_ NPs, Pd–SnO_2_ NPs, and Pt-SnO_2_ NPs. Additionally, O 1*s* peaks of noble metal-decorated SnO_2_ NPs were deconvoluted into three peaks, and the peak location is summarized in Table S2. Figure 4c displays the XPS spectra of Au, Pd, and Pt, which were conducted to investigate the surface chemical bonding state of noble metal NPs. The detailed deconvoluted peak positions are provided in Table S3. The presence of oxidized peaks in the noble metal decorated on SnO_2_ NPs revealed that the surface of the noble metals was partially oxidized, while the inner region remained in a metallic phase [59].

### Reducing Gas Sensing Properties of Metal-Decorated SnO_2_ NPs

**Fig. 5:**
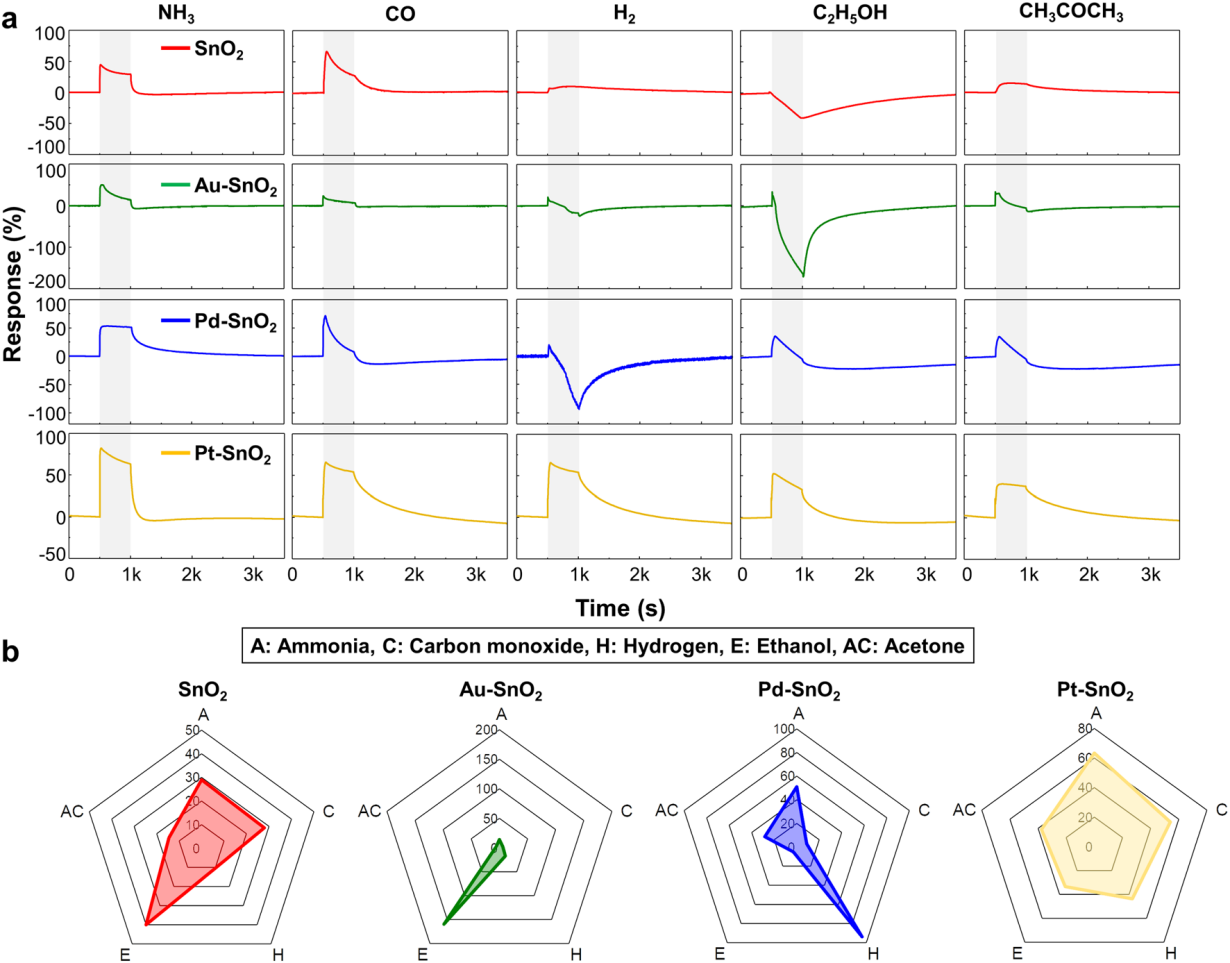
Gas responses of µLED-integrated gas sensor array to reducing gases.** a** Response curves and **b** polar plots of SnO_2_ NPs, Au-SnO_2_ NPs, Pd-SnO_2_ NPs, and Pt-SnO_2_ NPs to NH_3_, CO, H_2_, C_2_H_5_OH, and CH_3_COCH_3_

The NO_2_ sensing properties of Au-SnO_2_ NPs, Pd-SnO_2_ NPs, and Pt-SnO_2_ NPs were investigated under light illumination, as shown in Fig. S22. In comparison with SnO_2_ NPs, the response to 5 ppm of NO_2_ showed a decrease in Au-SnO_2_ NPs, Pd-SnO_2_ NPs, and Pt-SnO_2_ NPs, with the most significant decrease observed in Pt-SnO_2_ NPs. Various reducing gases including NH_3_, CO, H_2_, C_2_H_5_OH, and CH_3_COCH_3_ were investigated for SnO_2_ NPs, Au-SnO_2_ NPs, Pd-SnO_2_ NPs, and Pt-SnO_2_ NPs, as shown in Fig. 5a. The response of reducing gases denoted as *R*_r_ was defined using Eq. (8), which represents the response of reducing gases as the ratio of the resistance between air and gas atmosphere.

8 $$R_{{\text{r}}} = \, \left( {R_{{\text{g}}} - R_{{\text{a}}} } \right)/R_{{\text{g}}} \quad \cdot{1}00\,\left( \% \right)$$

Here, *R*_g_ and *R*_a_ are the same as Eq. (2). All sensors showed distinct selectivity patterns under blue light illumination. As shown in Fig. S23, the sensors were exposed to 4 repetitive pulses of reducing gases, exhibiting high reliability and repeatability. For a better comparison, the polar plots of SnO_2_ NPs, Au-SnO_2_ NPs, Pd-SnO_2_ NPs, and Pt-SnO_2_ NPs to reducing gases were obtained, as shown in Fig. 5b. SnO_2_ NPs exhibited moderate response to NH_3_, CO, and C_2_H_5_OH, Au-SnO_2_ NPs exhibited high sensitivity and selectivity toward C_2_H_5_OH, Pd-SnO_2_ NPs showed a good response to H_2_, and Pt-SnO_2_ NPs exhibited a good response to several gases, especially NH_3_. The response tendency of reducing gases under various light intensities was measured and is detailed in Fig. S24. The gas mixtures containing 50 ppm of NH_3_, H_2_, and C_2_H_5_OH were measured by SnO_2_ NPs, Au-SnO_2_ NPs, Pd-SnO_2_ NPs, and Pt-SnO_2_ NPs, which exhibited distinct patterns of dynamic curves, as shown in Fig. S25. Overall, noble metal-decorated SnO_2_ NPs-based 2 × 2 gas sensor array demonstrated the ability to discriminate various gases under light illumination.

Based on the conventional mechanism of chemoresistive gas sensors, the reaction with reducing gases leads to a decrease in the resistance of n-type SnO_2_. However, the gas response curves of our gas sensor array showed different patterns compared to conventional gas sensors. These unique sensing patterns can be explained by the oxygen species on the surface of SnO_2_ NPs. Oxygen molecules exist as ionized oxygen species on the surface of SnO_2_ NPs, especially in the form of O_2_^−^ at room temperature [60]. When a reducing gas such as CH_3_COCH_3_ is exposed to SnO_2_, the stable form of O_2_^−^ is less reactive with CH_3_COCH_3_ [61]. Thus, for the reaction to occur, it is assumed that O_2_^−^ dissociates into O^−^ before reacting with CH_3_COCH_3_, which increases the resistance, as shown in Eq. (9). After that, the reaction of CH_3_COCH_3_ with O^−^ decreases the resistance, as shown in Eq. (10).

9 $${\text{O}}_{{2{\text{(ad)}}}}^{ - } + \, e^{ - } \to \, 2{\text{O}}^{ - }_{{\text{(ad)}}}$$

10 $${\text{CH}}_{{3}} {\text{COCH}}_{{{3}({\text{g}})}} + {\text{ 8O}}^{ - }_{{({\text{ad}})}} \to {\text{ 3CO}}_{{{2}({\text{g}})}} + {\text{ 3H}}_{{2}} {\text{O}}_{{({\text{g}})}} + {\text{ 8e}}^{ - }$$

To supplement this mechanism, the dynamic response shape of SnO_2_ NPs to CH_3_COCH_3_ was measured under various conditions of temperature, light, and atmosphere, as shown in Fig. S26. When CH_3_COCH_3_ was introduced to SnO_2_ NPs in the dark state at room temperature, the resistance increased because oxygen dissociation was more dominant than the decomposition of CH_3_COCH_3_. As the temperature increased, the resistance variation for the input of CH_3_COCH_3_ showed opposite direction, resulting in a decrease of the resistance. The elevated temperature activates the adsorbed oxygen and decreases the activation energy for the reaction with CH_3_COCH_3_, making the decomposition of CH_3_COCH_3_ more dominant than oxygen dissociation. At 150 °C, the tendency for resistance to decrease with the input of CH_3_COCH_3_ was abruptly enhanced because O^−^ becomes the stable oxygen species at 150 °C, and is reactive with reducing gases [60]. In the case of light illumination on SnO_2_ NPs, the resistance also increased with input of CH_3_COCH_3_ at room temperature. However, the response and recovery were faster than in the dark state due to the light-activated oxygen species, which is more reactive than adsorbed oxygen, as shown in Eqs. (11) and (12) [4].

11 $${\text{O}}_{2(hv)}^{ - } + \, e^{ - } \to \, 2{\text{O}}^{ - }_{(hv)}$$

12 $${\text{CH}}_{{3}} {\text{COCH}}_{{{3}({\text{g}})}} + {\text{ 8O}}^{ - }_{{({\text{hv}})}} \to {\text{ 3CO}}_{{{2}({\text{g}})}} + {\text{ 3H}}_{{2}} {\text{O}}_{{({\text{g}})}} + {\text{ 8e}}^{ - }$$

As the temperature increases with light illumination, the tendency of dynamic curve shape was similar to that in dark state. The elevated temperature causes the resistance to decrease with reaction of CH_3_COCH_3_. To clarify the effect of oxygen, CH_3_COCH_3_ was measured under N_2_ atmosphere. When CH_3_COCH_3_ was exposed to SnO_2_ NPs under N_2_ atmosphere, the resistance decreased. CH_3_COCH_3_ directly adsorbs SnO_2_ NPs and draws electrons due to the absence of oxygen [62]. Therefore, it can be assumed that the resistance increase in reducing gases at low temperature is due to oxygen dissociation of the stable oxygen species.

Moreover, noble metal decoration on SnO_2_ NPs results in distinct responses to various gases. In the case of SnO_2_ NPs, C_2_H_5_OH showed a decrease in resistance, while other gases showed an increase in resistance. It is assumed that the decomposition of C_2_H_5_OH is more dominant than oxygen dissociation, unlike other reducing gases. When Au is decorated on SnO_2_, Au promotes oxygen dissociation with chemical sensitization, which decreases the activation energy for the reaction of reducing gases [63, 64]. The catalytic effect of Au increases the decomposition of reducing gases, which suppresses the increase in resistance caused by oxygen dissociation on SnO_2_. Therefore, the reducing gases showed a decrease in response, but C_2_H_5_OH showed an increase in response. This leads Au-SnO_2_ NPs to exhibit good sensitivity and selectivity to C_2_H_5_OH. For Pd-SnO_2_ NPs, Pd shows chemical sensitization to H_2_ through phase transition of PdH_x_ [65]. The activation energy for the reaction between H_2_ and oxygen decreases due to the dissolution of H_2_ to H^+^ ion. Therefore, H_2_ reaction with oxygen is more dominant than oxygen dissociation, causing the resistance of Pd-SnO_2_ NPs to H_2_ to decrease. For Pt-SnO_2_ NPs, Pt accelerates oxygen dissociation on SnO_2_, which causes the resistance of all reducing gases resistance to increase. This results in Pt-SnO_2_ NPs showing high response for various reducing gases through an increase in resistance. Overall, the μLED platform was demonstrated to detect various reducing gases with tunable selectivity.

### Real-Time Gas Monitoring with a Fully Hardware-Integrated E-Nose Array

**Fig. 6:**
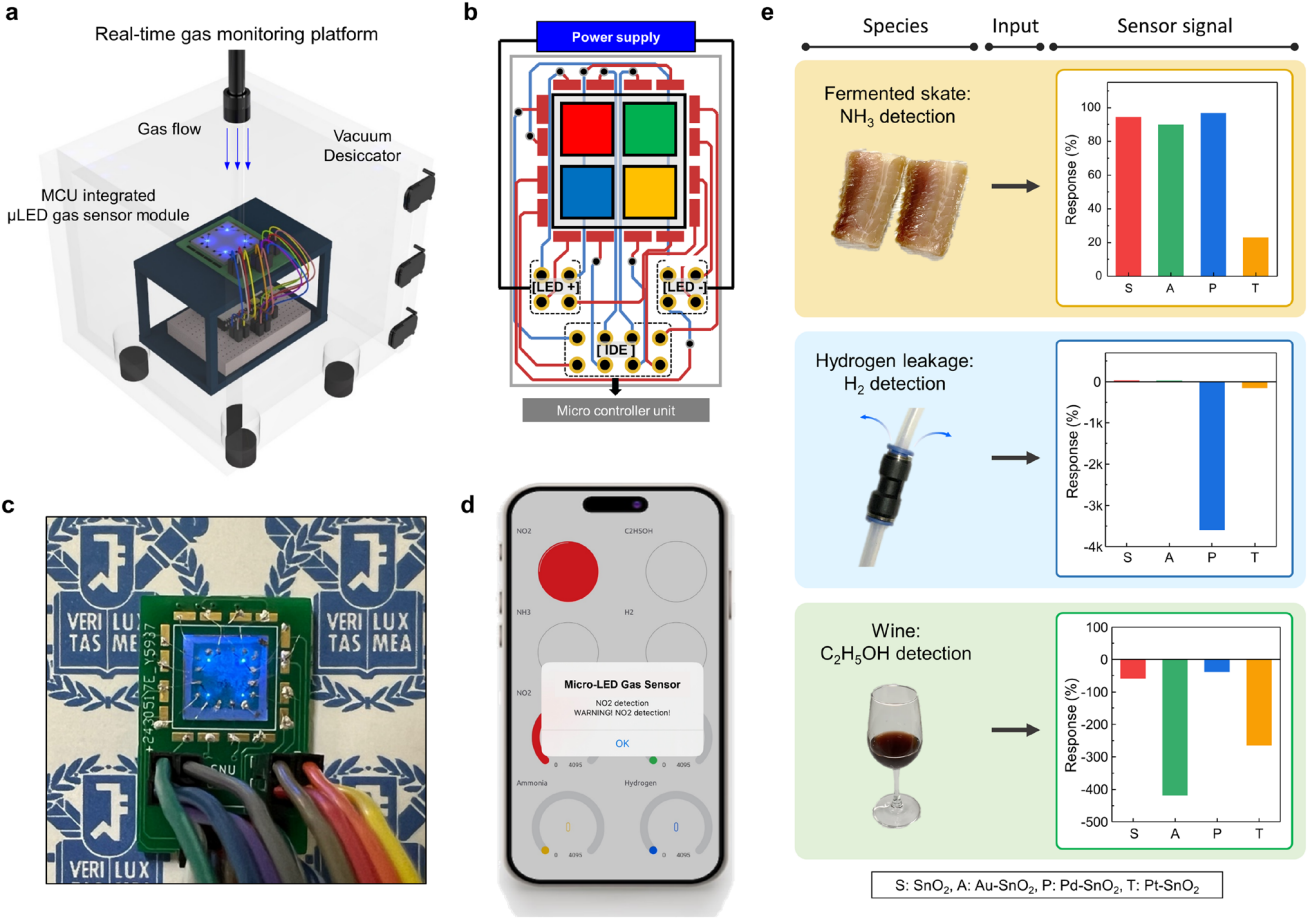
Real-time gas monitoring using a mobile application. **a** Schematic illustration of real-time gas sensing platform setup. **b** Optical image of MCU-integrated µLED gas sensor module. **c** A schematic illustration of PCB circuit design. **d** User interface of the mobile gas notification application when the module selectively detects NO_2_ gas. **e** Response plots of the practical application of gas sensor array to detect fermented skate, hydrogen leakage, and wine

Real-time gas monitoring of the μLED gas sensor array was tested to verify its suitability for daily use, as shown in Figs. 6a and S27, and Video S1. The device was connected to a PCB via a Pt-wire-bonding process for operation, as shown in Fig. 6b. The MCU provided the 3.3 V operation voltage to the μLEDs, and the 0.5 V sensing voltage to each sensing component to monitor the responses simultaneously and independently. Figure 6c shows the customized design of PCB, which ensured the μLEDs deliver separate electrical signals in response to different target gases. The device is connected to a mobile phone via Wi-Fi for gas hazard notification. The MCU is programmed to send a signal when the resistance change induced by the gas reaction exceeds a predetermined threshold, as shown in Fig. S28 and Note S3. If the target gas concentration is over the threshold value, the signal is transmitted, and the corresponding response value appears in the mobile application. The application activates color-coded alarms, displays warning text messages, and monitors the gas response from each sensor. Figure 6d shows the user interface of the mobile application when the sensor detects NO_2_ gas. By utilizing these platforms, µLED gas sensor array can identify the distinct responses of each sensor and compare their selectivity via wireless communication. Moreover, the practical applications of µLED gas sensor array were demonstrated by detecting a fermented skate (Raja kenojei), hydrogen leakage, and wine, as shown in Fig. 6e and Video S2. Fermented skate is associated with NH_3_, which is produced by microbial activity through the degradation of urea and trimethylamine oxide [66]. Detecting hydrogen leakage in gas pipes is crucial for the prevention of hydrogen explosion, which has the possibility of ignition above 4% of H_2_ [67]. Wine, as an alcoholic beverage, contains C_2_H_5_OH. When the different species were introduced to the gas sensor array, the sensor signals showed distinct responses, as shown in Fig. S29. In conclusion, the real-time detection of distinct 4 gases and practical application to 3 different species by µLED gas sensor array demonstrated the potential capability for establishing advanced e-nose platforms.

## Conclusion

The μLED-integrated SnO_2_ NPs gas sensor array is capable of selectively detecting various gases at room temperature while operating at extremely low power levels. Fabrication of the 20 × 20 μm^2^ sized μLED gas sensor platform was conducted via a conventional semiconductor device fabrication process. By integrating μLEDs with the gas sensor, blue light activation of sensing materials with micro-watt power consumption was achieved. The 1.5 μm gap between the light source and the sensing materials maximizes the light activation efficiency. The μLED-integrated SnO_2_ NPs gas sensor exhibited a high response to 5 ppm of NO_2_ (6928) with a power consumption of 63.2 μW. The volcano shape of gas response, depending on the light intensity, was interpreted using FDTD simulation of light absorbance. For selective detection of reducing gases, noble metals such as Au, Pd, and Pt were decorated onto SnO_2_ NPs. With the catalytic effect of the noble metals, the noble metal-decorated SnO_2_ NPs gas sensor array showed a distinct pattern in response to reducing gases. Real-time gas monitoring was achieved by connecting the gas sensor to a mobile phone via Wi-Fi using an MCU. Overall, this work is expected to contribute to the enlargement of μLED gas sensor fields and diversify the detectable gases for our healthy living environment.

## Supplementary Information

Below is the link to the electronic supplementary material.

Supplementary file 1 (DOCX 3584 KB)

Supplementary file 2 (mp4 34448 KB)

Supplementary file 3 (mp4 34448 KB)
