# Supplementary material for: Real-Time Tunable Gas Sensing Platform Based on SnO2 Nanoparticles Activated by Blue Micro-Light-Emitting Diodes
Source: Nanomicro Lett. 2024 Aug 8;16:261. doi: 10.1007/s40820-024-01486-2 (PMC11306663; doi:10.1007/s40820-024-01486-2)
Supplement: Supplementary file 1 — (DOCX 3584 KB) [file 40820_2024_1486_MOESM1_ESM.docx]

Supporting Information for

Real-Time Tunable Gas Sensing Platform Based on SnO_2_ Nanoparticles Activated by Blue Micro-Light-Emitting Diodes

Gi Baek Nam^1, +^, Jung-El Ryu^1, 2, +^, Tae Hoon Eom^1, +^, Seung Ju Kim^1, 3, +^, Jun Min Suh^1, 2^, Seungmin Lee^1^, Sungkyun Choi^1^, Cheon Woo Moon^4^, Seon Ju Park^1^, Soo Min Lee^1^, Byungsoo Kim^1^, Sung Hyuk Park^1^, Jin Wook Yang^1^, Sangjin Min^5^, Sohyeon Park^1^, Sung Hwan Cho^1^, Hyuk Jin Kim^1^, Sang Eon Jun^1^, Tae Hyung Lee^1^, Yeong Jae Kim^1^, Jae Young Kim^1^, Young Joon Hong^6^, Jong-In Shim^5^, Hyung-Gi Byun^7^, Yongjo Park^8^, Inkyu Park^9,^ *, Sang-Wan Ryu^10,^ *, and Ho Won Jang^1,8,^ *

^1^Department of Materials Science and Engineering, Seoul National University, Seoul 08826, Republic of Korea

^2^Department of Mechanical Engineering, Research Laboratory of Electronics, Massachusetts Institute of Technology, Cambridge, MA, 02139, USA

^3^Ming Hsieh Department of Electrical and Computer Engineering, University of Southern California, Los Angeles, CA, USA

^4^Department of Display Materials Engineering, Soonchunhyang University, Asan 31538, Republic of Korea

^5^Department of Photonics and Nanoelectronics, BK21 FOUR ERICA-ACE Center, Hanyang University ERICA, Ansan 15588, Republic of Korea

^6^Department of Nanotechnology and Advanced Materials Engineering, Sejong University, Seoul 05006, Republic of Korea

^7^Department of Electronics, Information and Communication Engineering, Kangwon National University, Samcheok 25913, Republic of Korea

^8^Advance Institute of Convergence Technology, Seoul National University, Suwon 16229, Republic of Korea

^9^Department of Mechanical Engineering, Korea Advanced Institute of Science and Technology, Daejeon 34141, Republic of Korea

^10^Department of Physics, Chonnam National University, Gwangju 500-757, Republic of Korea

*^+^* Gi Baek Nam, Jung-El Ryu, Tae Hoon Eom, and Seung Ju Kim contributed equally to this work

*Corresponding authors. E-mail: [hwjang@snu.ac.kr](mailto:hwjang@snu.ac.kr) (Ho Won Jang), [sangwan@chonnam.ac.kr](mailto:sangwan@chonnam.ac.kr) (Sang-Wan Ryu), [inkyu@kaist.ac.kr](mailto:inkyu@kaist.ac.kr) (Inkyu Park)

**Supplementary Figures and Tables**

**
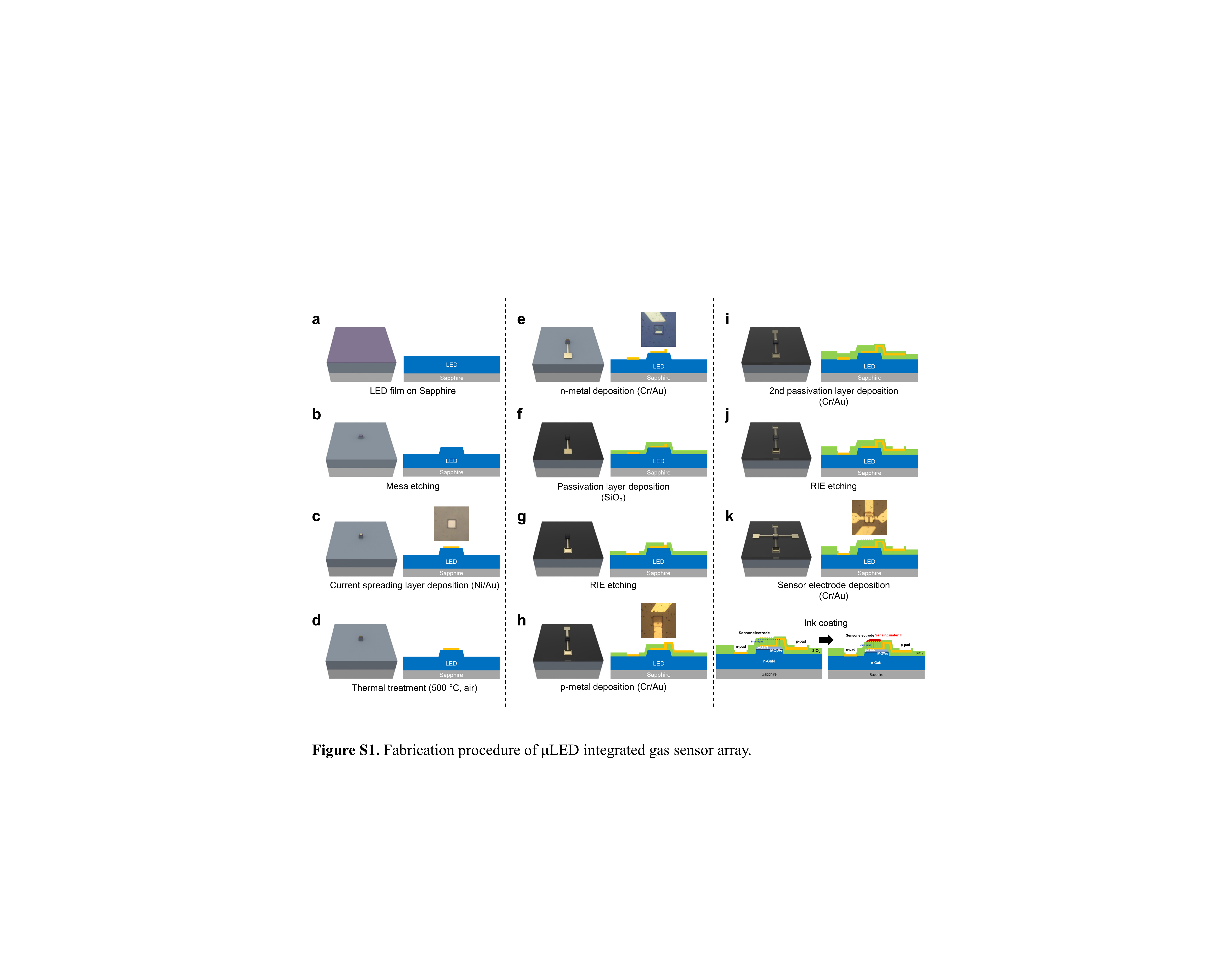
**

**Fig. S1** Fabrication procedure of μLED integrated gas sensor array

**
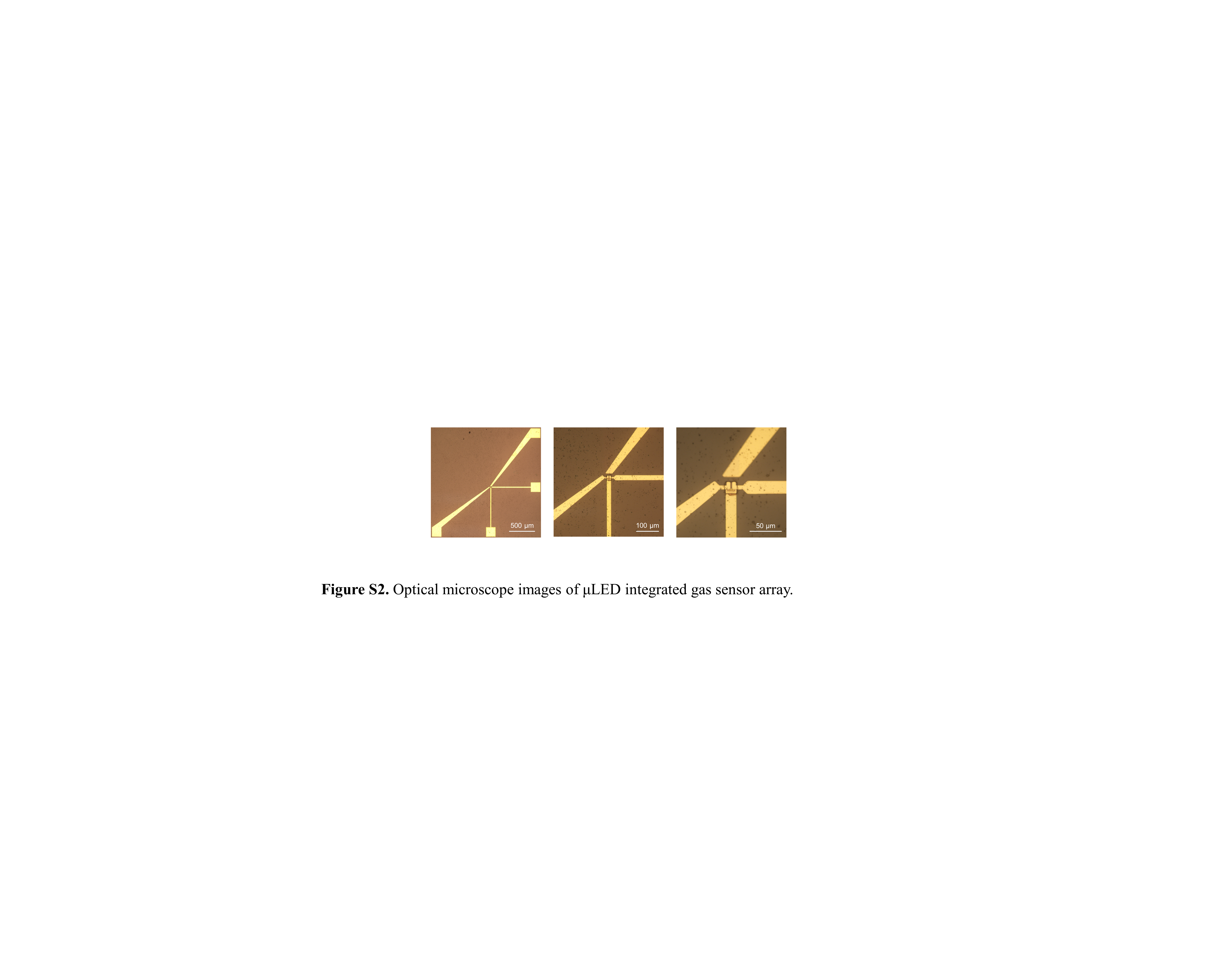
 Fig. S2** Optical microscope images of μLED integrated gas sensor array


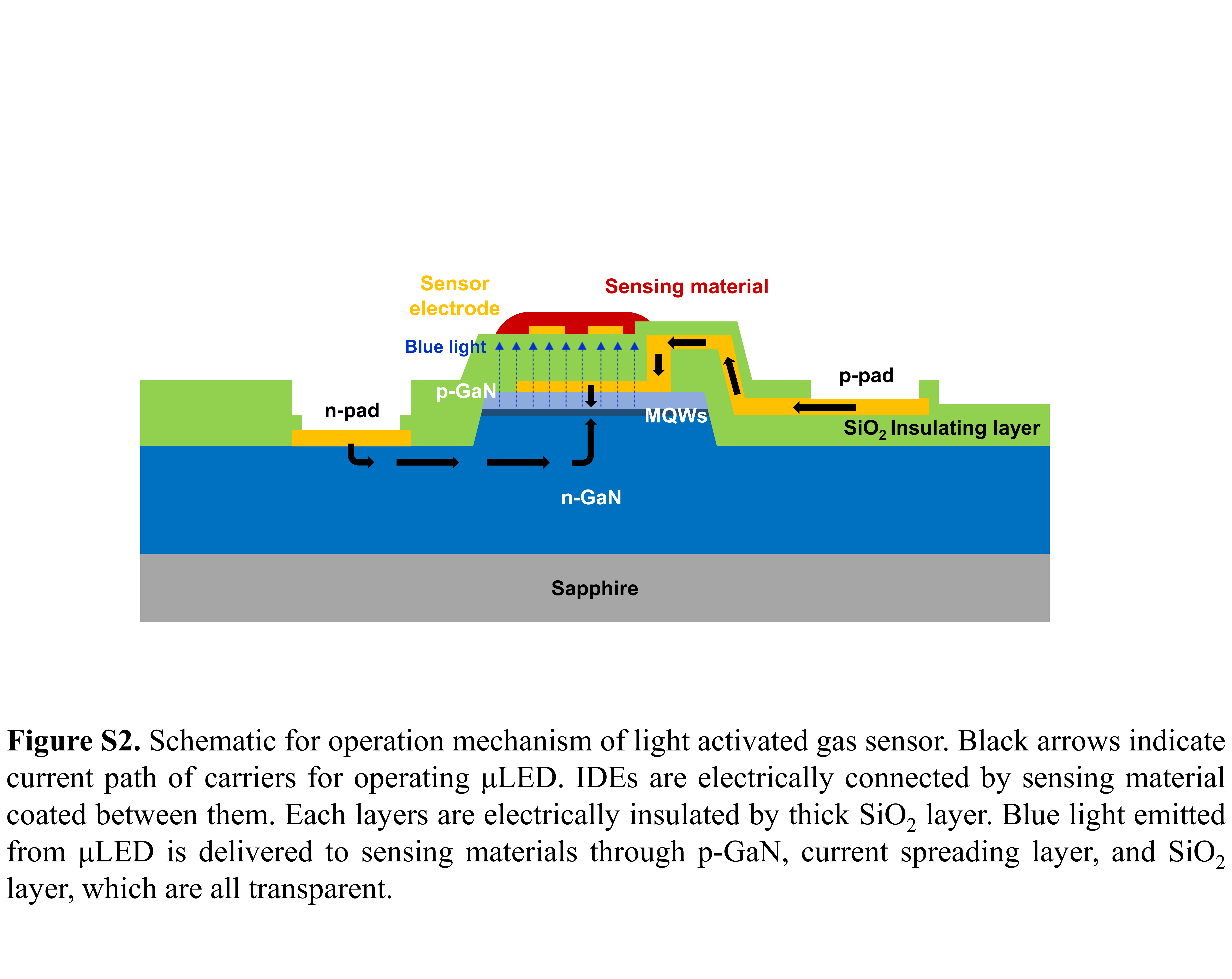


Fig. S3 Schematic for operation mechanism of light-activated gas sensor. Black arrows indicate current path of carriers for operating μLED. Sensor electrodes are electrically connected by sensing material coated between them. Each layer is electrically insulated by thick SiO_2_ layer. Blue light emitted from μLED is delivered to sensing materials through p-GaN, current spreading layer, and SiO_2_ layer, which are all transparent

**
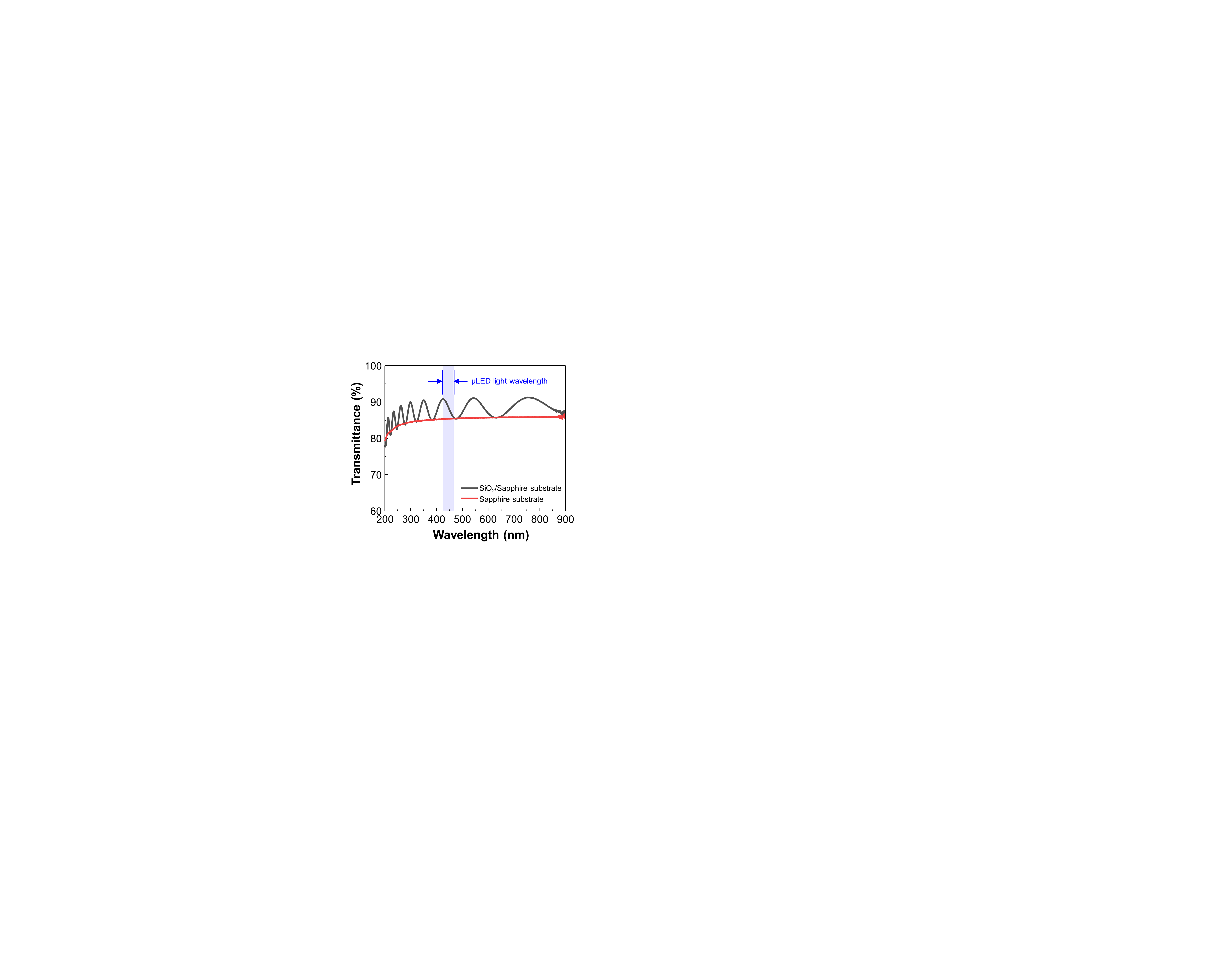
**

**Fig. S4** Transmittance of double-side polished (DSP) sapphire substrate and 700 nm of SiO_2_ film on DSP sapphire by UV-Vis spectroscopy. The wave of transmittance in the SiO_2_ film on sapphire is caused by the difference in reflective index between SiO_2_ and sapphire substrate.[S1] The wavelength of μLED light was indicated by a blue line in the graph.


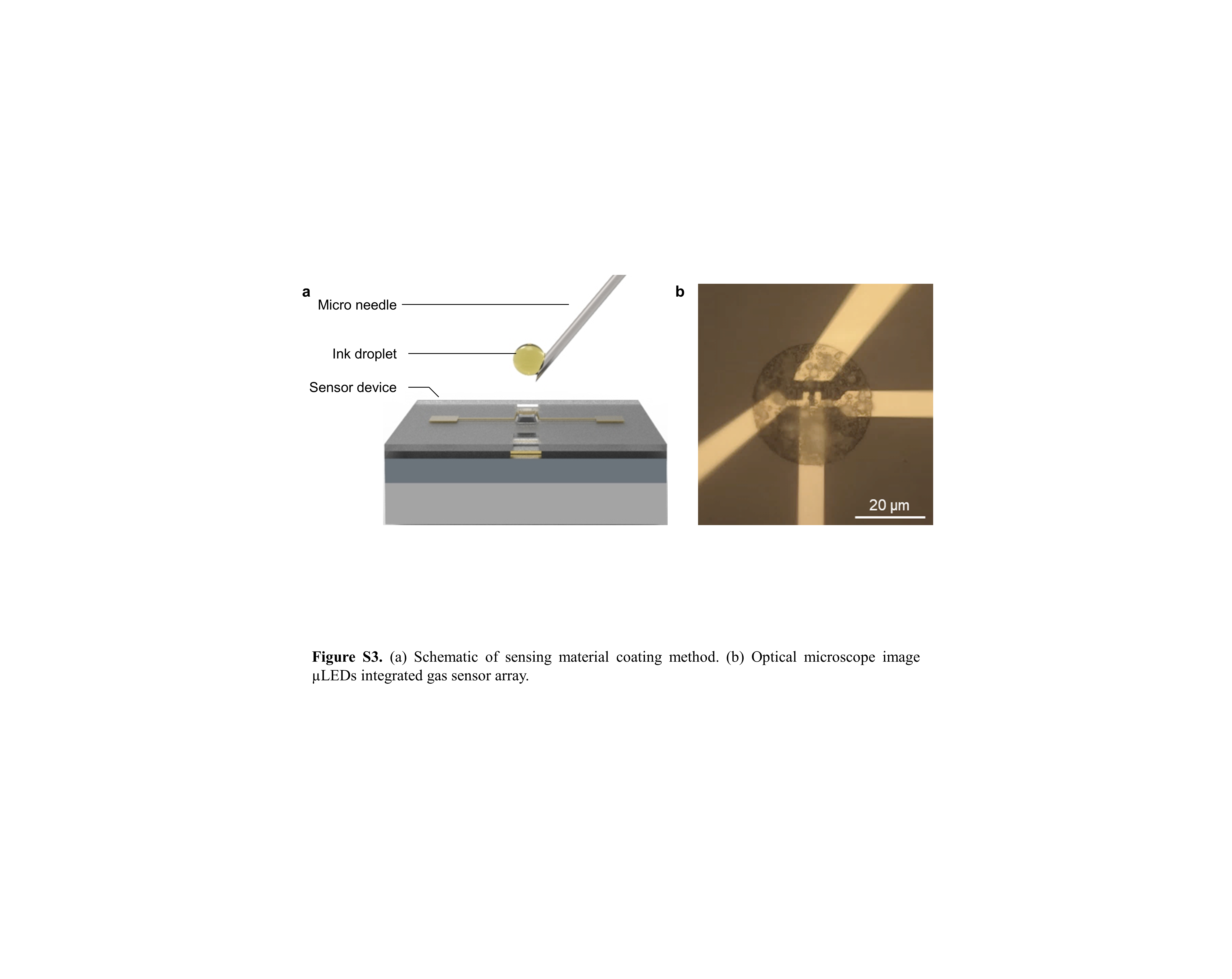
 **Fig. S5 a** Schematic of sensing material coating method. **b** Optical microscope image of µLEDs integrated gas sensor array


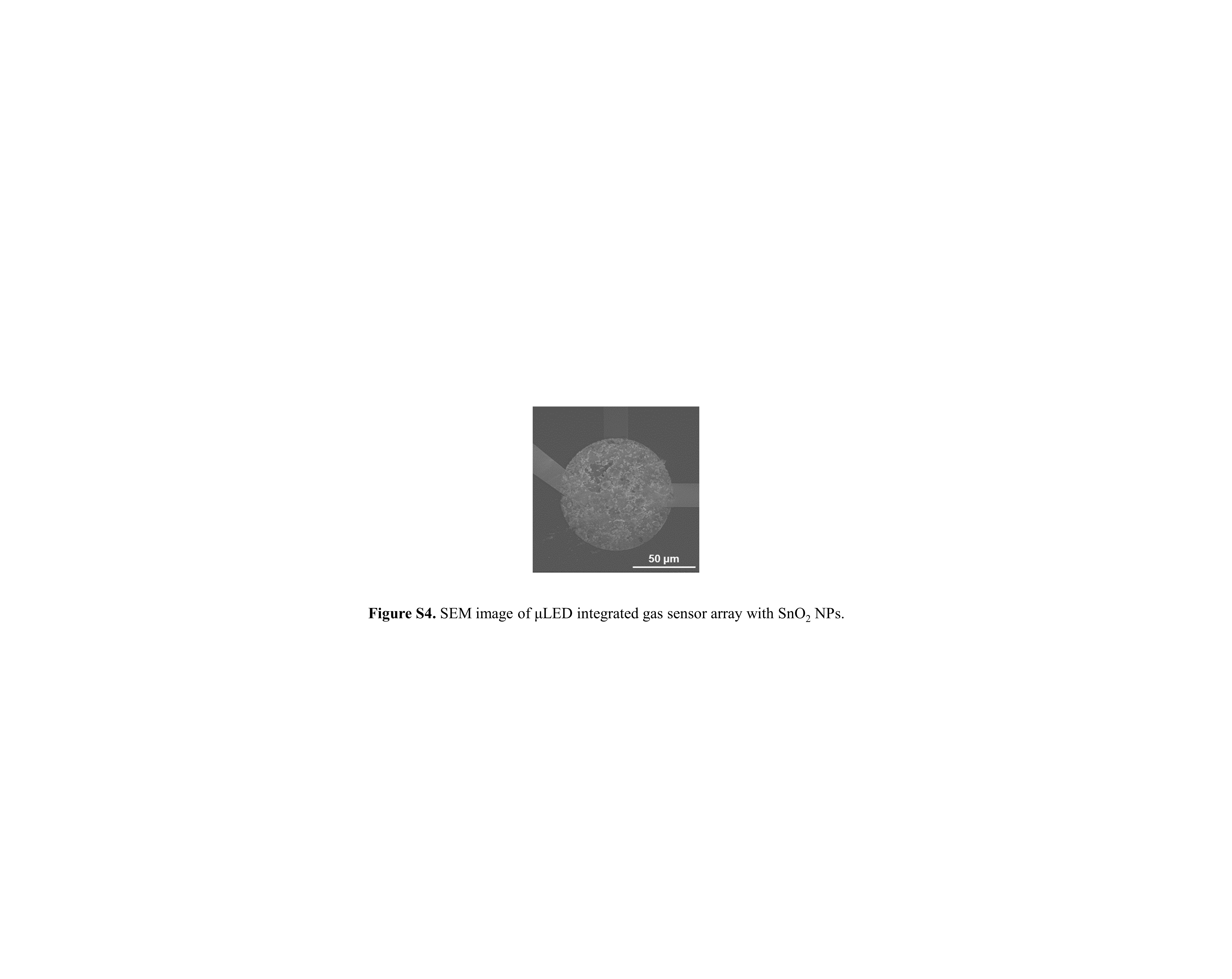


**Fig. S6** SEM image of μLED integrated gas sensor array with SnO_2_ NPs


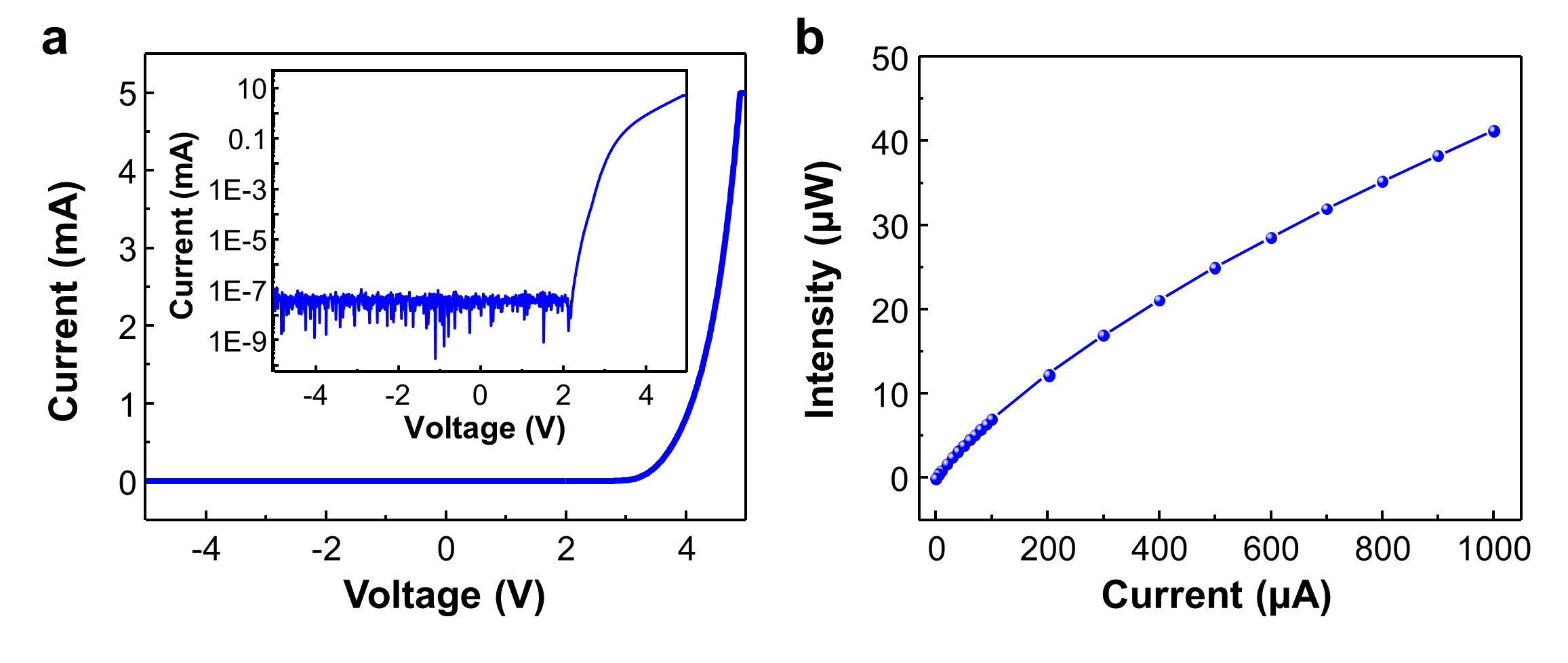
 **Fig. S7** **a** I-V characteristic curve of µLEDs. The inset shows semi-log plot of the I-V curve. **b** Light intensity plot of µLEDs according to injection current


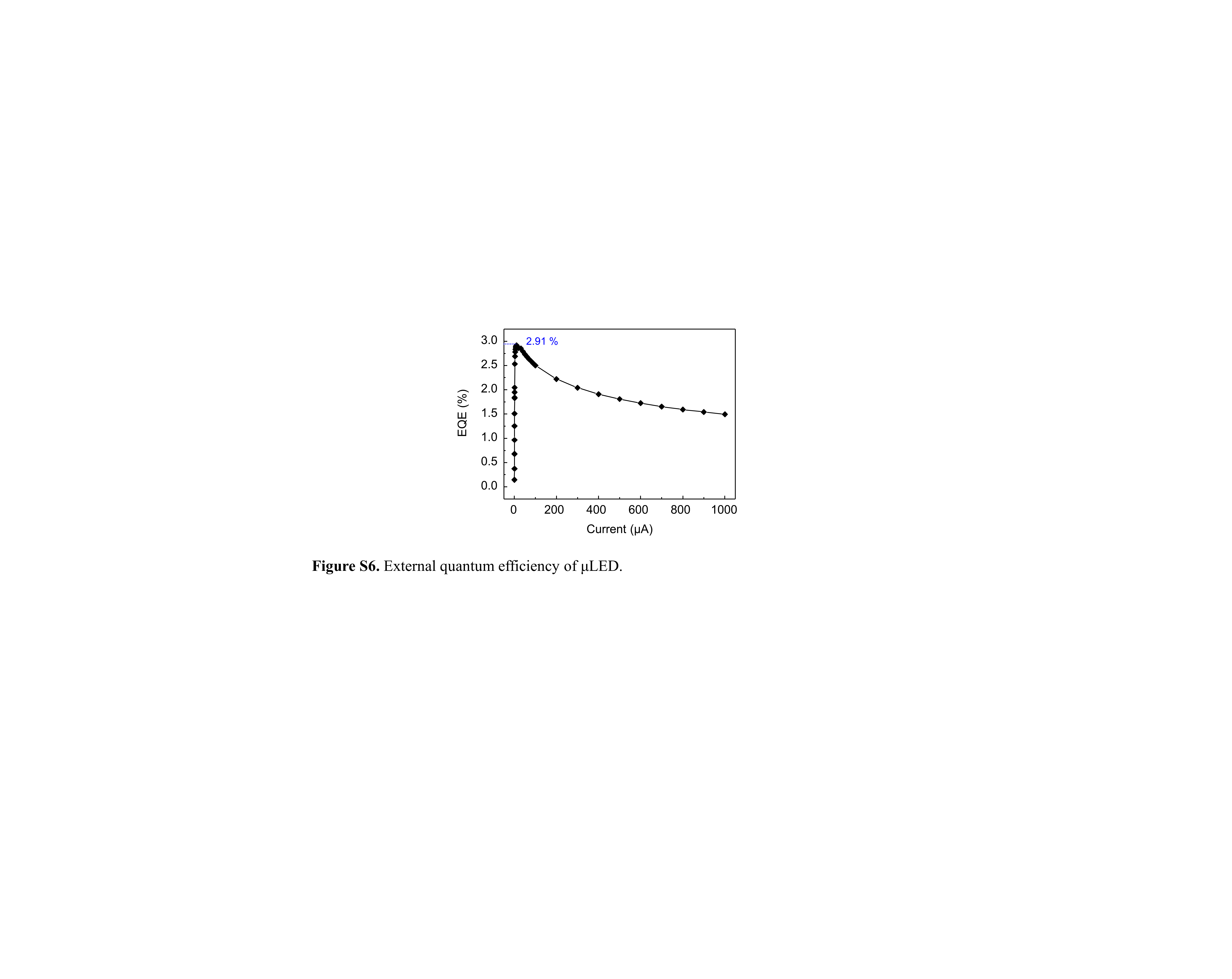


**Fig. S8** External quantum efficiency of μLED


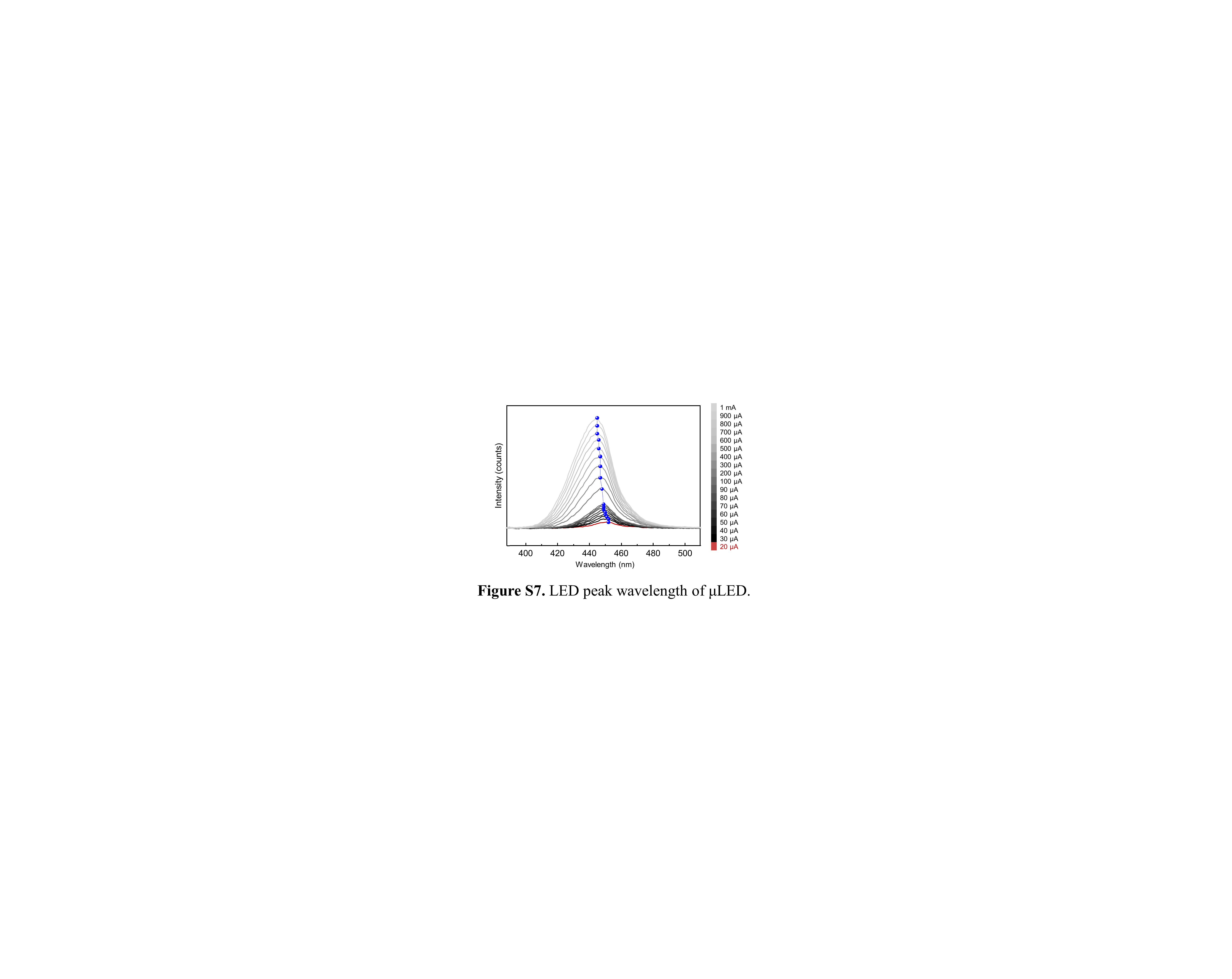


**Fig. S9** LED peak wavelength of μLED


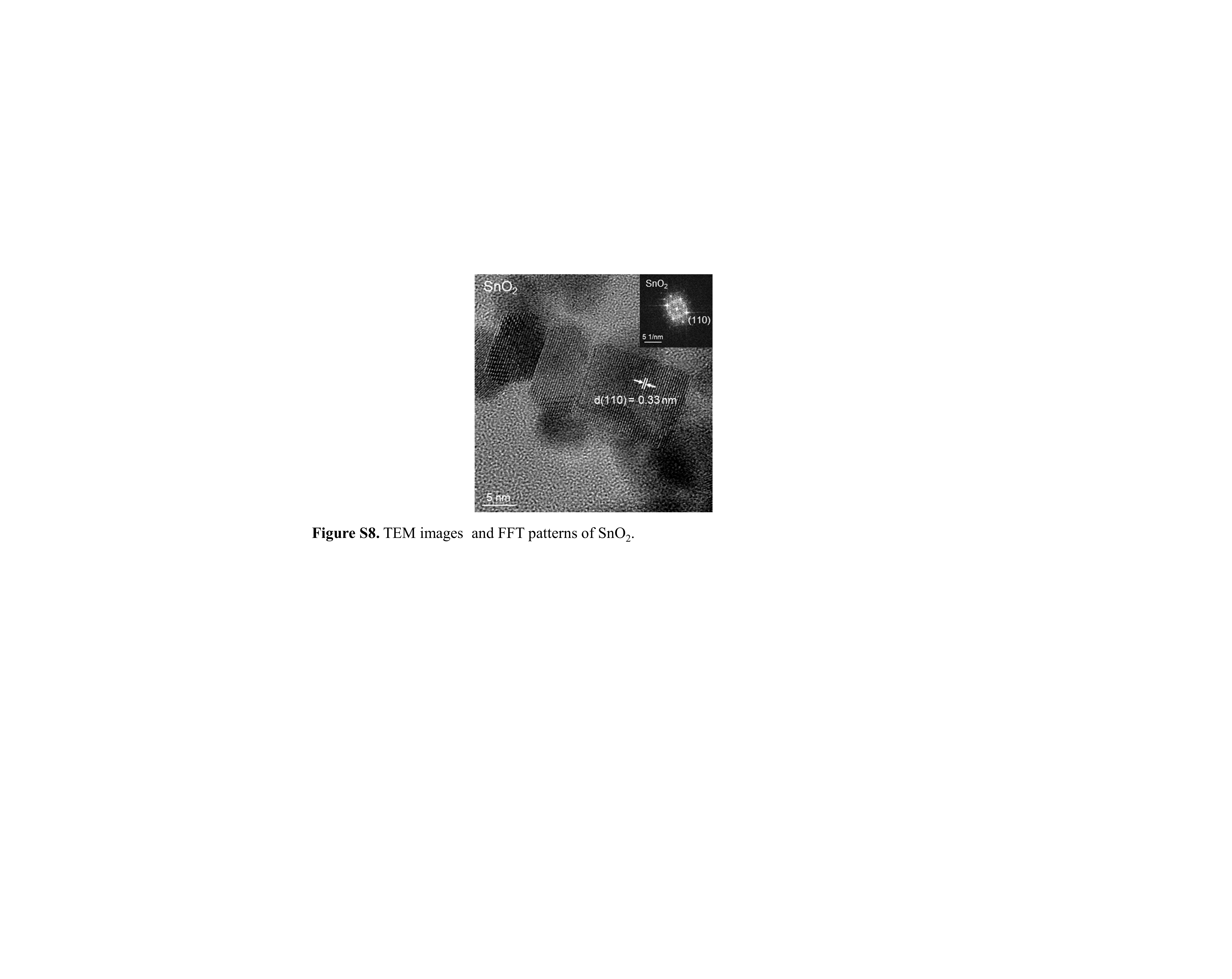


**Fig. S10** TEM image and FFT patterns of SnO_2_ NPs


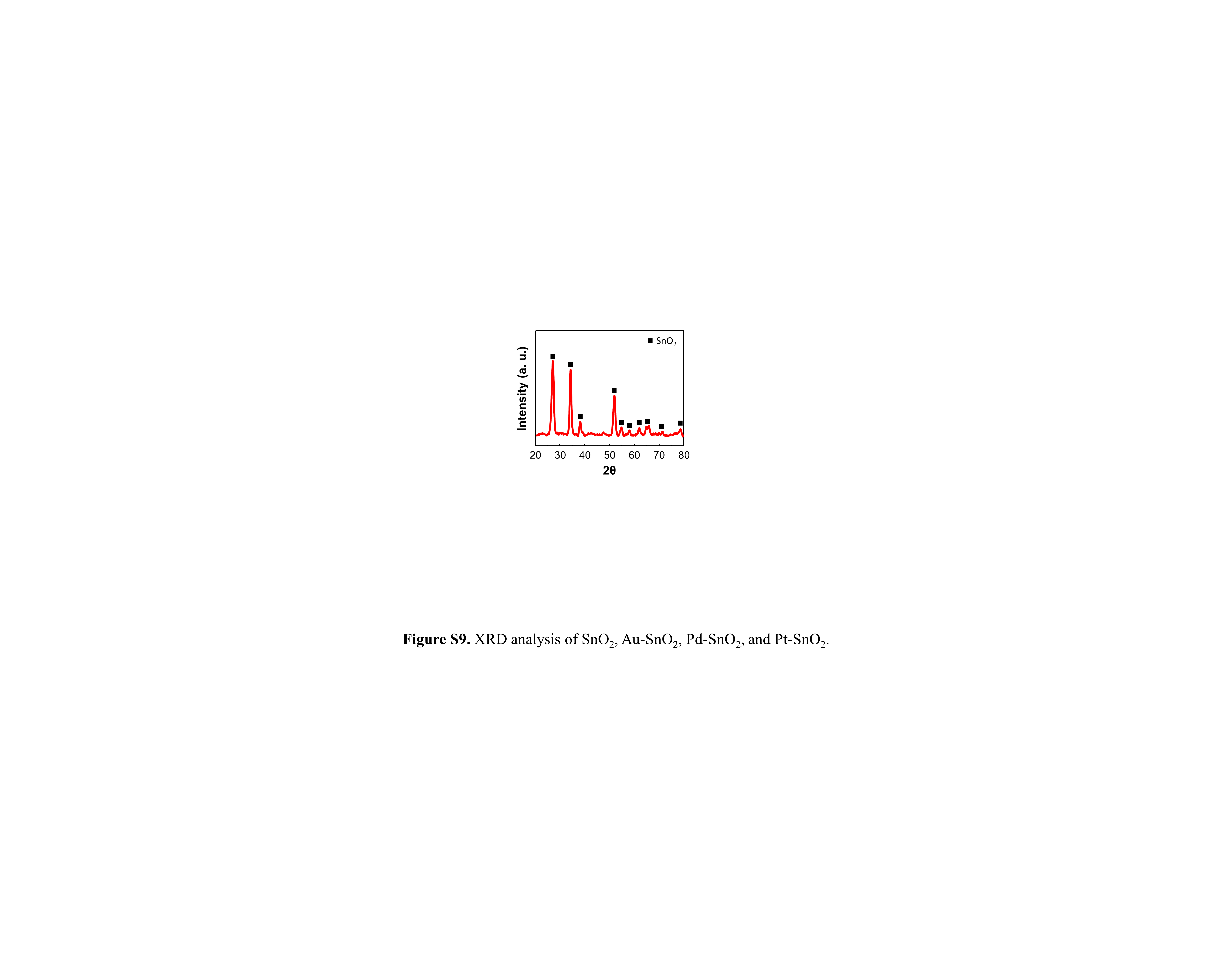


**Fig. S11** XRD spectrum of SnO_2_ NPs


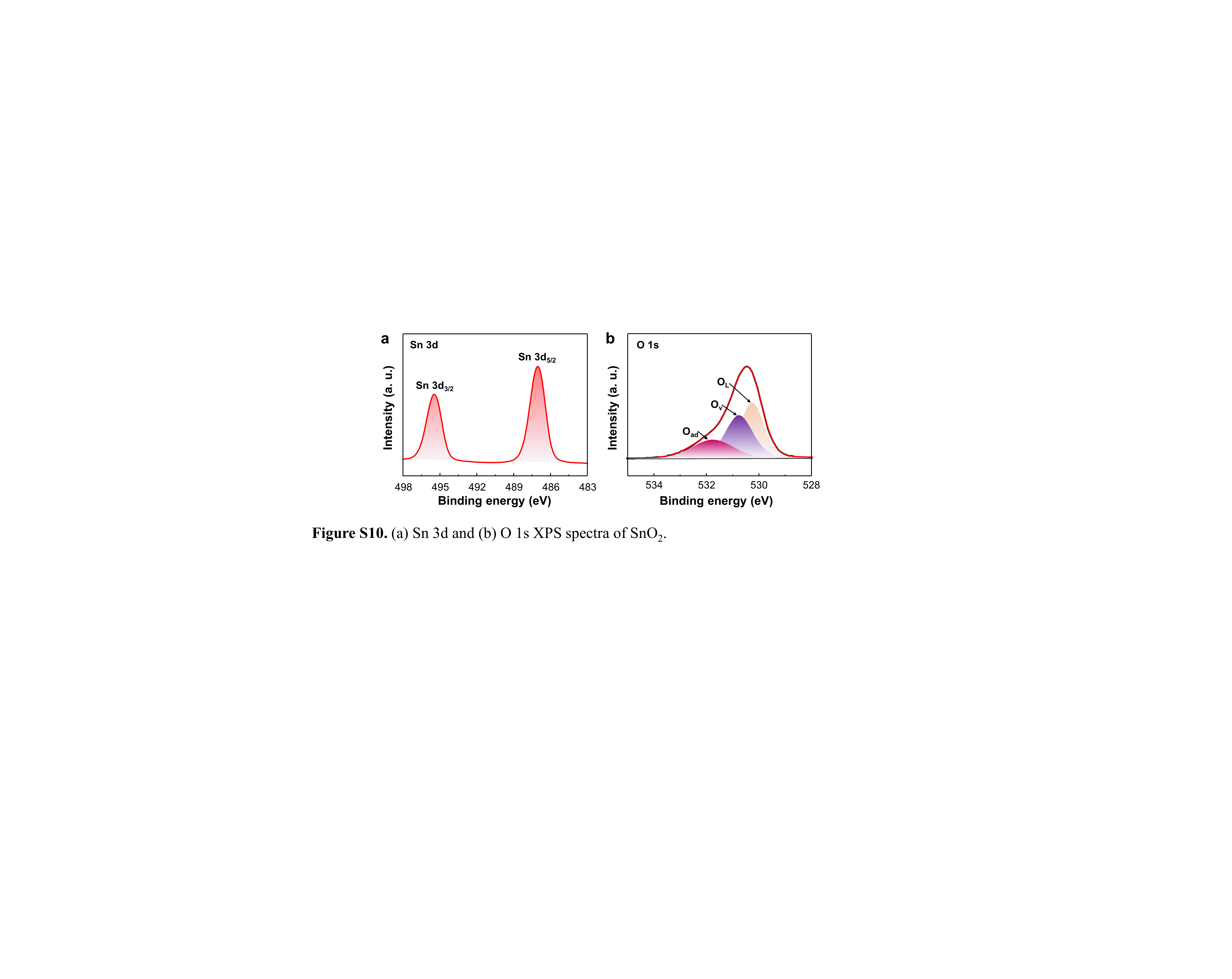


**Fig. S12** **a** Sn 3d and **b** O 1s XPS spectra of SnO_2_ NPs

*
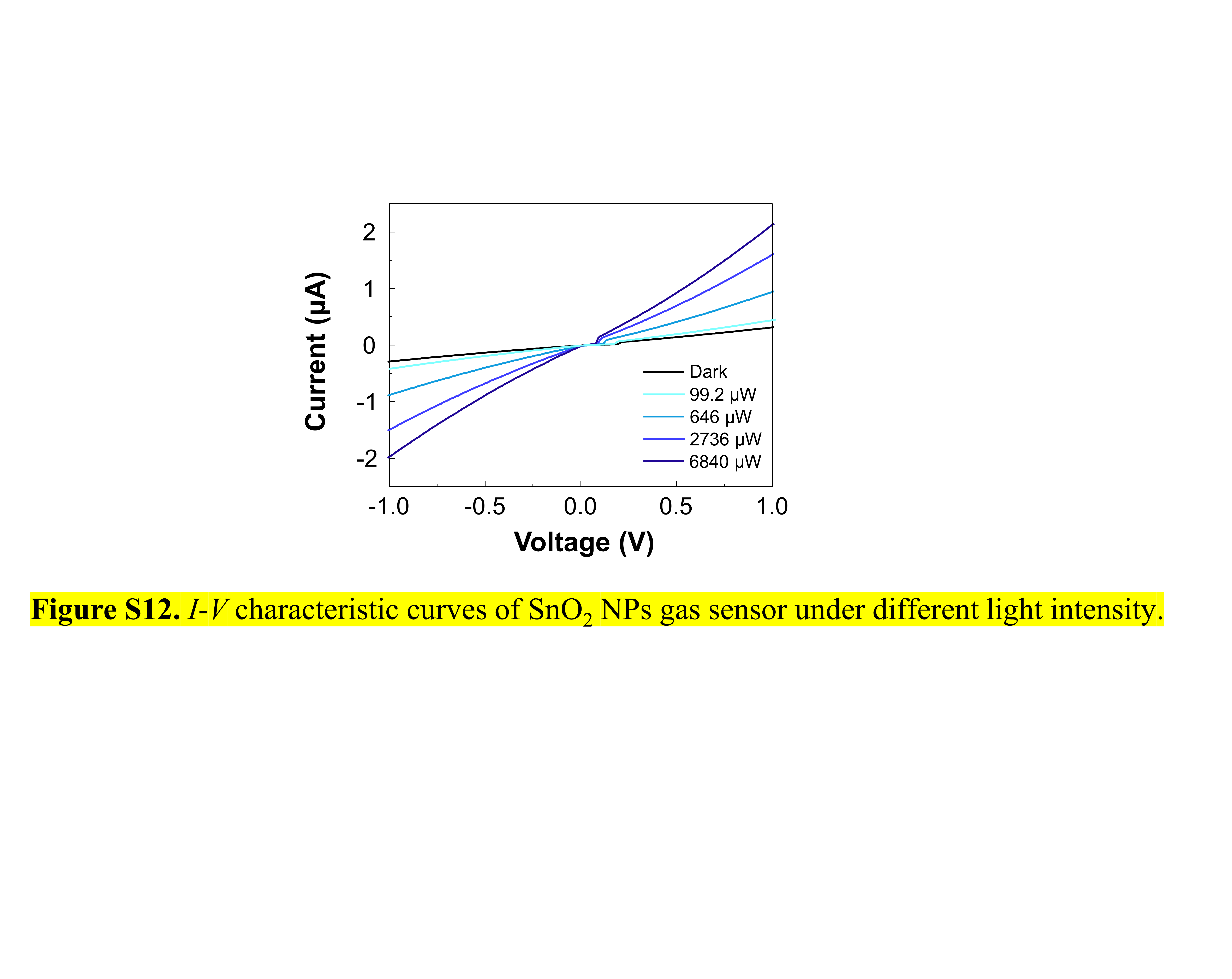
*

Fig. S13 *I*-*V* characteristic curves of SnO_2_ NPs gas sensor under different light intensities

**
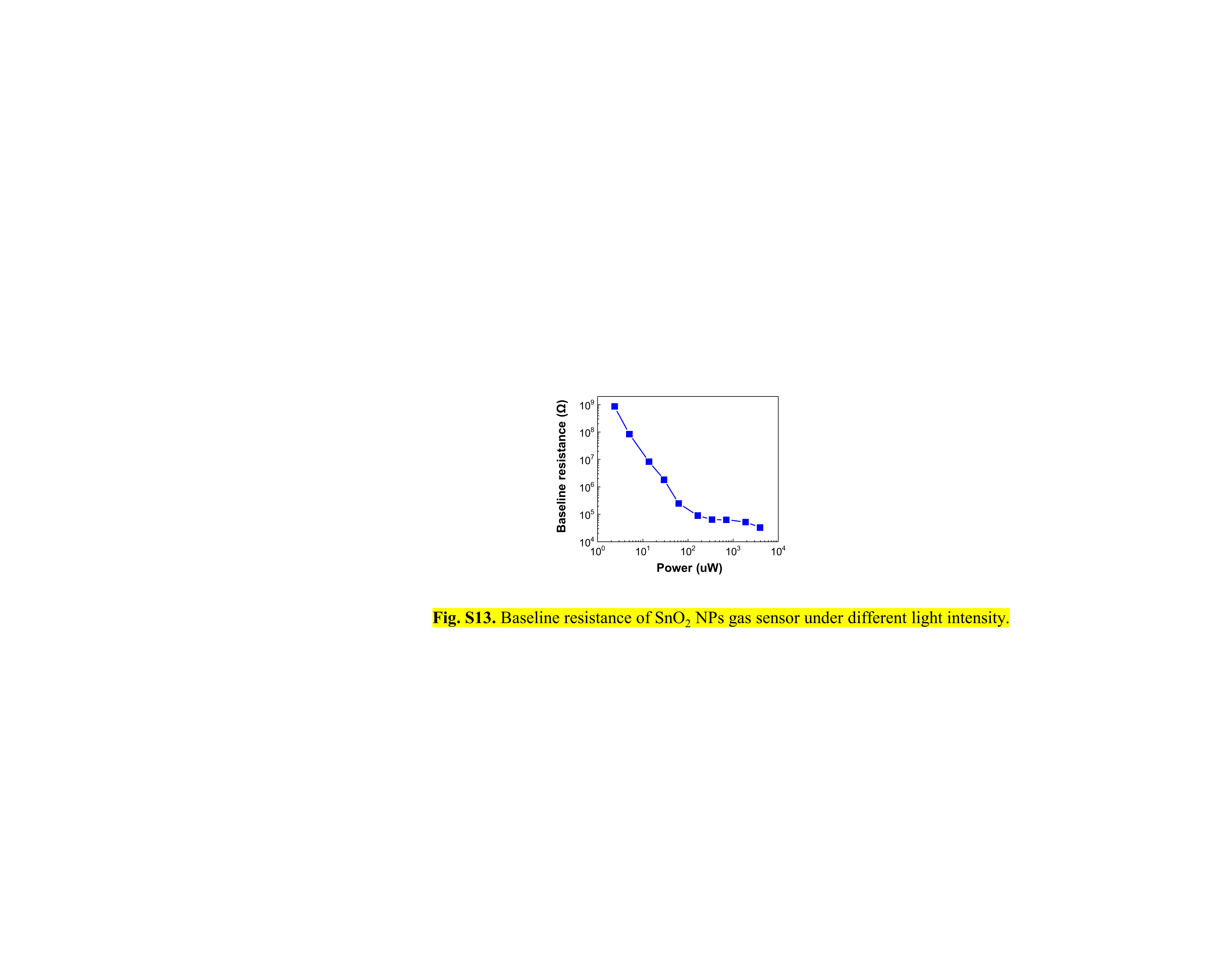
**

**Fig. S14** Baseline resistance of SnO_2_ NPs gas sensor under different light intensities

**Note S1 Mechanism of light-activated SnO_2_ gas sensor**

Under ambient air condition, oxygen molecules are adsorbed onto the surface of SnO_2_ NPs. The adsorbed oxygens draw electrons from the SnO_2_ NPs surface, generating a depletion layer at the surface, as shown in equation (S1). At room temperature, O_2_^-^ is a dominant form among the oxygen species including O^-^ and O^2-^ [S2].

O_2(g)_ + e^-^ → O_2_^-^_(ad)_ (S1)

When NO_2_ is introduced to SnO_2_ NPs without light irradiation, NO_2_ is difficult to react with SnO_2_ NPs due to the presence of adsorbed oxygen which is in a stable form of O_2_^-^.[S3] For NO_2_ reaction, NO_2_ molecules compete with adsorbed oxygen to react with SnO_2_ NPs.[S4] However, the presence of stable oxygen inhibits NO_2_ to trap electrons from SnO_2_ NPs at room temperature. In contrast, blue light irradiation enhances the reaction of NO_2_ molecules with SnO_2_ NPs by photo-generating electron and hole pairs in SnO_2_ [S5]. The photo-generated holes react with adsorbed oxygen and desorb it, as shown in equation (S3) [S6]. The photo-generated electrons combine with oxygen in the air to produce photo-activated oxygen, as shown in equation (S4). The photo-activated oxygen molecules are more reactive and loosely bound to SnO_2_ compared to adsorbed oxygen molecules under dark condition [S7].

hν → e^-^_(photo)_ + h^+^_(photo)_ (S2)

h^+^_(photo)_ + O_2­_^-^ _(ad)_ → O_2(g)_ (S3)

O_2(g)_ + e^-^_(photo)_ → O_2_^-^_(photo)_ (S4)

When NO_2_ is introduced to SnO_2_ under light illumination, NO_2_ molecules can easily take over the active sites at the surface of SnO_2_ NPs due to the desorption of stable oxygen, capturing the photo-generated electrons from the conduction band, as shown in equation (S5).[8] Moreover, photo-activated oxygen, which is more reactive than stable oxygen, reacts with NO_2_ and transfers electrons to NO_2_ molecules, as shown in equation (S6).

NO_2(g)_ + e^-^_(photo)_ → NO_2_^-^_(ad)_ (S5)

NO_2(g)_ + O_2_^-^_(photo)_ → NO_2_^-^_(ad)_ + O_2(g)_ (S6)

At the recovery stage, photogenerated holes react with adsorbed NO_2_ molecules and desorb them, enabling SnO_2_ NPs to recover at room temperature, as shown in equation (S7).

NO_2_^-^_(ad)_ + h^+^_(photo)_ ↔ NO_2(g)_ (S7)

The described light activation mechanism demonstrates that the NO_2_ gas reaction is reversible at room temperature.


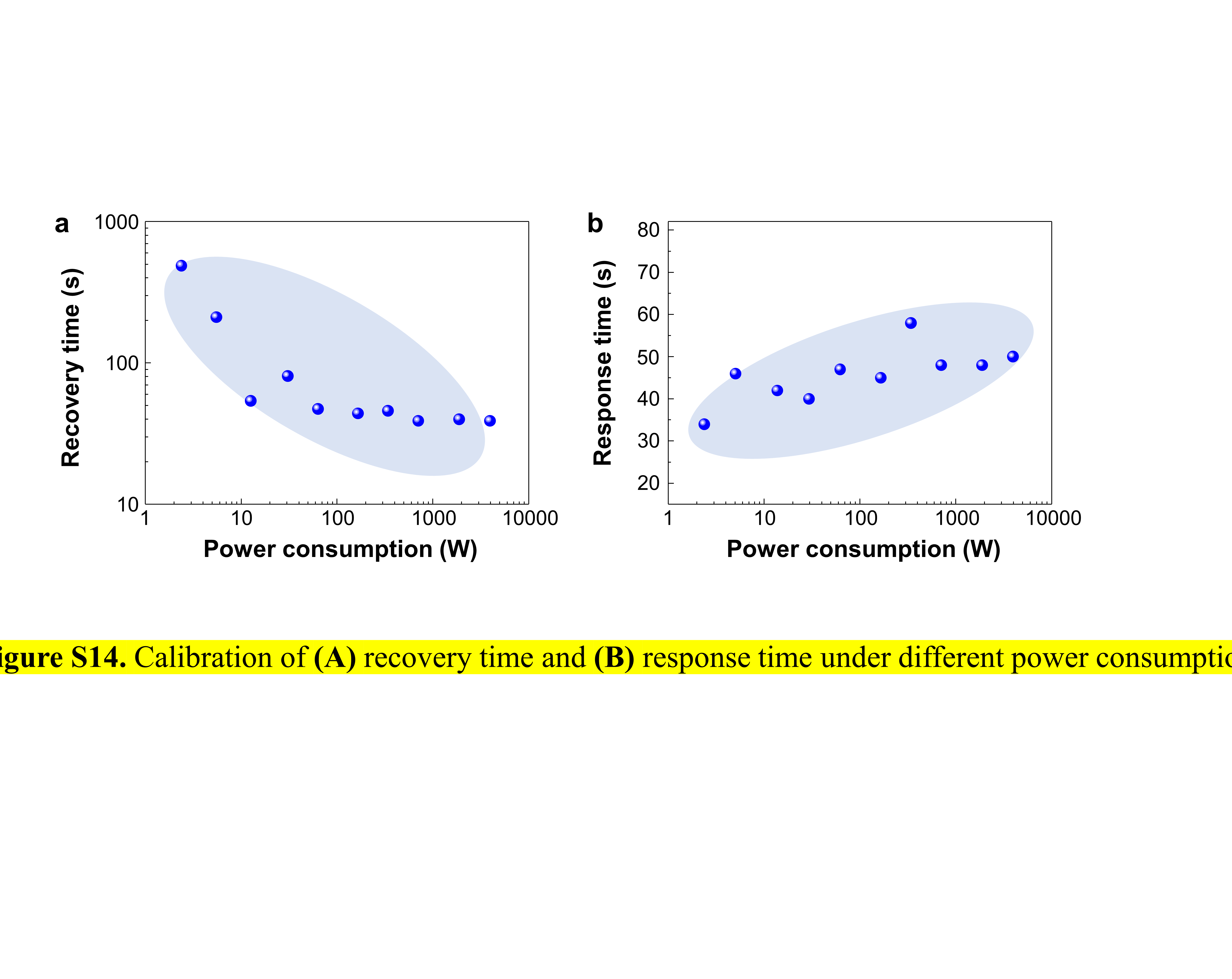
 **Fig. S15** Calibration of **a** recovery time and **b** response time under different power consumption


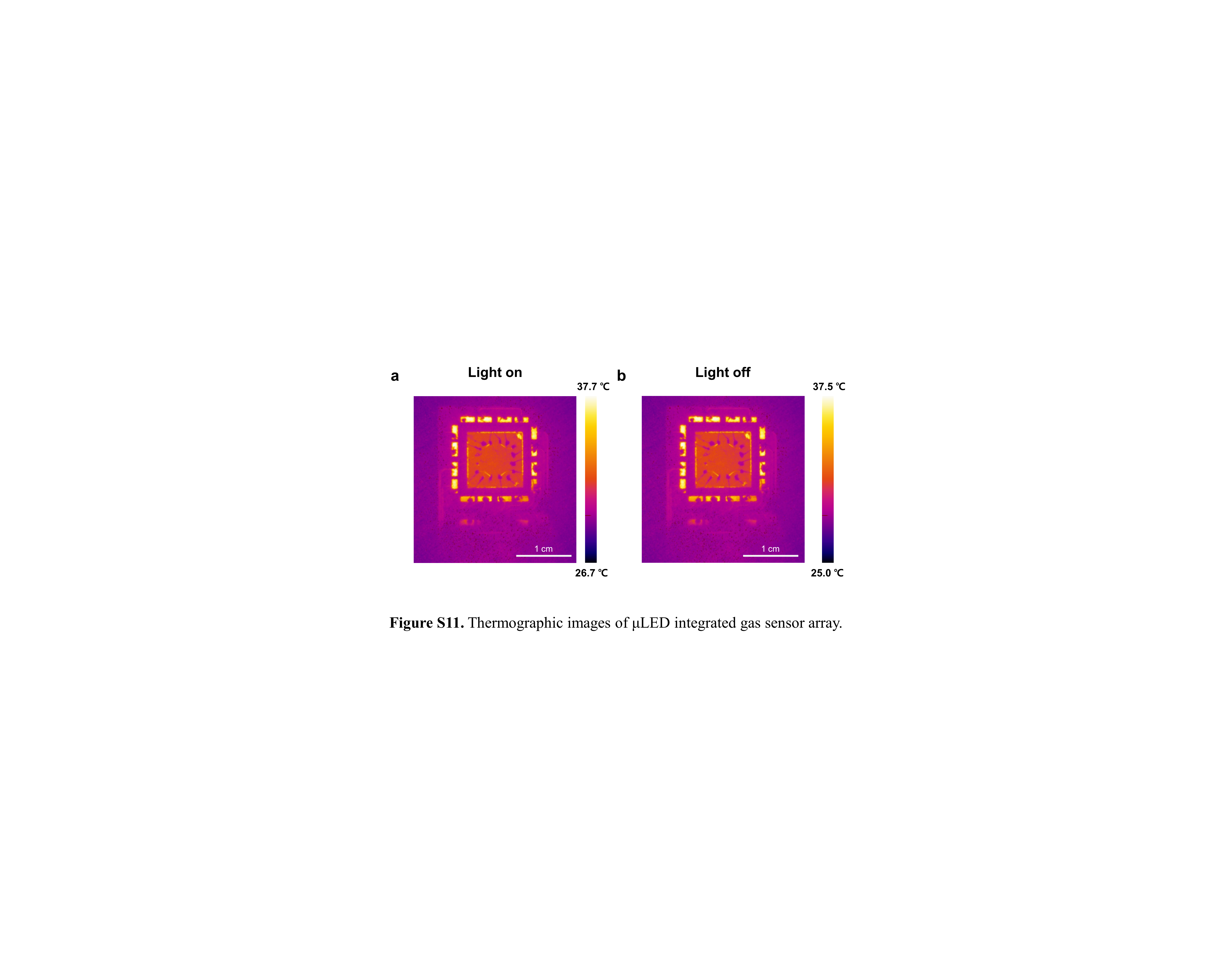


**Fig. S16** Thermographic images of μLED integrated gas sensor array for **a** light-on state with a power of 176 µW to each μLED and **b** light-off state

**Note S2 Material characteristics of SnO_2_ NPs contributing to enhancement factor of gas sensors**

The gas sensing performance of SnO_2_ NPs in material characteristics can be explained by three factors: ⅰ) utility factor, ⅱ) receptor function, and ⅲ) transducer function. The utility factor is related to the diffusion of gas into sensing materials. SnO_2_ NPs are arranged in a random stack on μLED, forming a porous structure that facilitates the diffusion of gas molecules from the upper surface to the bottom layer of the SnO_2_ NPs. Therefore, most SnO_2_ NPs can participate in the reaction with gas, which leads to an increase in reactivity. The receptor function is related to the gas reactivity with adsorbed oxygen on surface of SnO_2_ NPs. The high surface-to-volume ratio of SnO­_2_ NPs provides plenty of active sites for the gas reaction, and the numerous oxygen vacancies in SnO_2_ NPs enhance the gas reactivity [S9] The transducer function is associated with the change in charge density due to gas reactions, which affects electrical conductivity. SnO_2_ NPs with a size of 10 nm appear to be composed of nano-sized grains. As the depletion regions, which are generated by adsorbed oxygen, occupy the entire grain area of SnO_2_ NPs, the conduction channel of SnO_2_ NPs almost disappears [S10]. The majority of electrons are trapped on the surface of SnO_2_ NPs, which leads to a steep change of carrier concentration in gas reaction. In this state, resistance variation by gas reaction is inversely proportional to the size of grains [S11]. Thus, the nano-size grains in SnO_2_ NPs increase the response dramatically. The enhanced gas sensing properties of SnO_2_ NPs at room temperature under blue light illumination can be attributed to the aforementioned three factors.

**
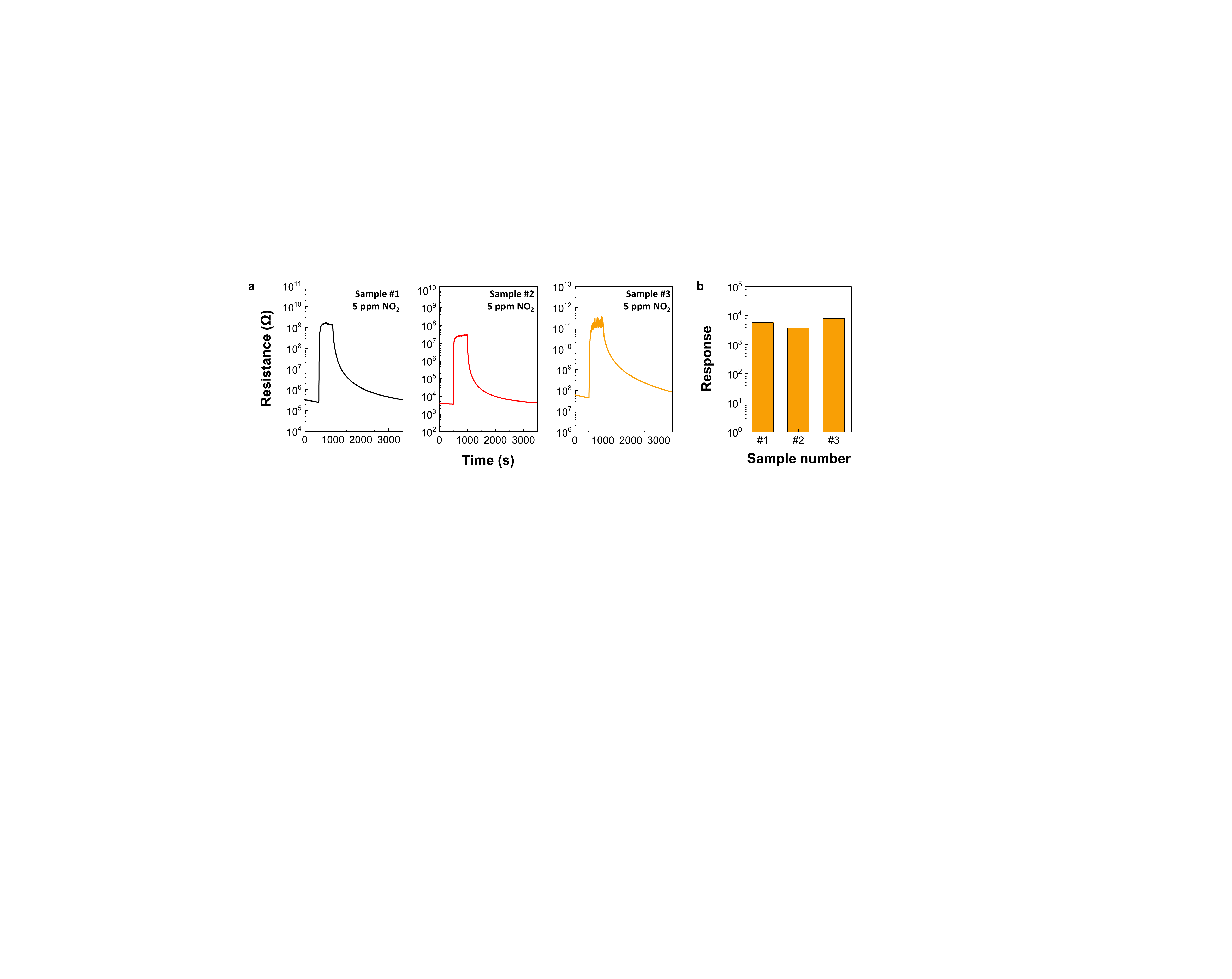
 Fig. S17** **a** Dynamic curves and **b** response of SnO_2_ NPs to 5 ppm of NO_2_ across various samples to confirm device uniformity


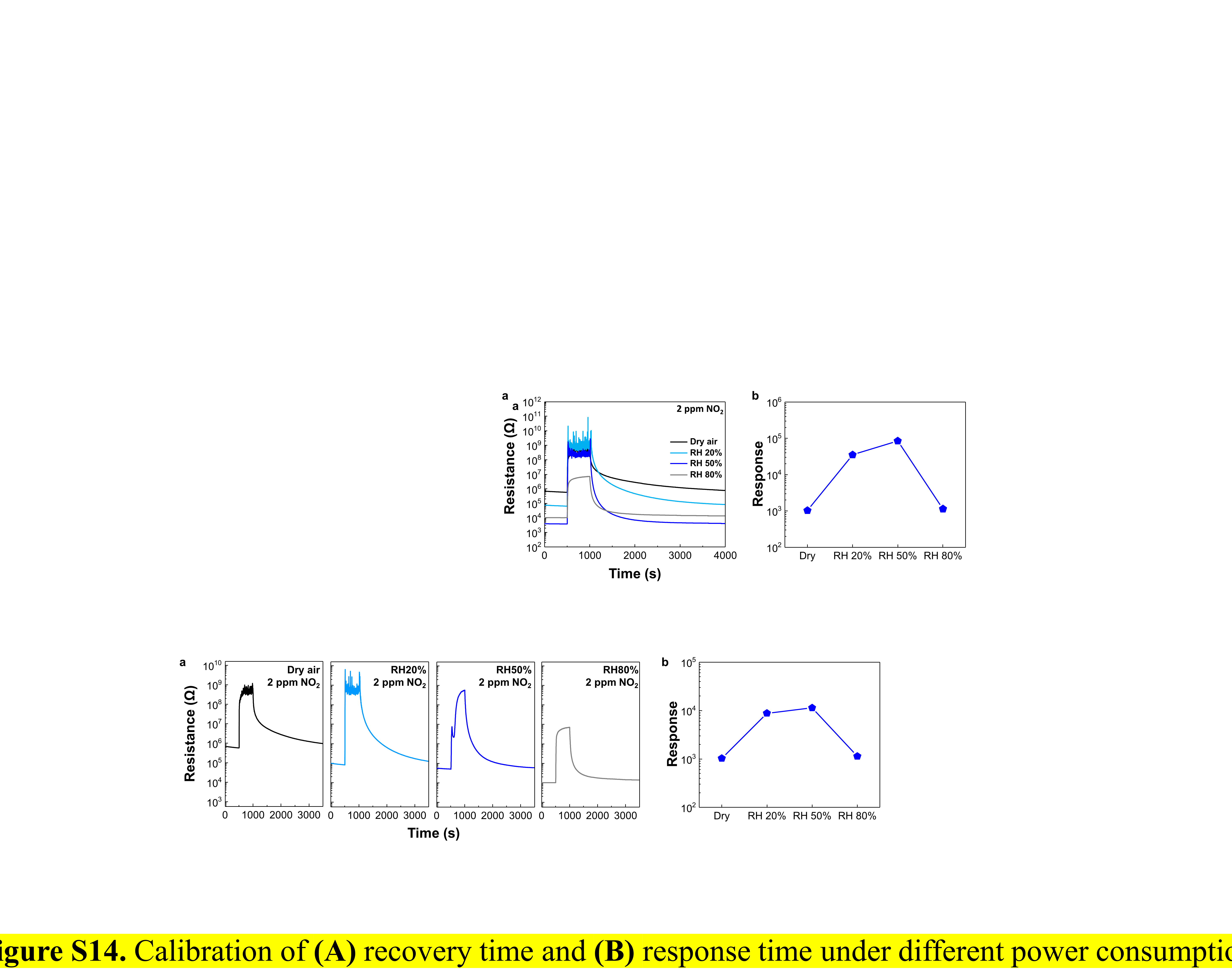
 **Fig. S18** **a** Dynamic curves and **b** response of SnO_2_ NPs to 2 ppm of NO_2_ under various humid conditions


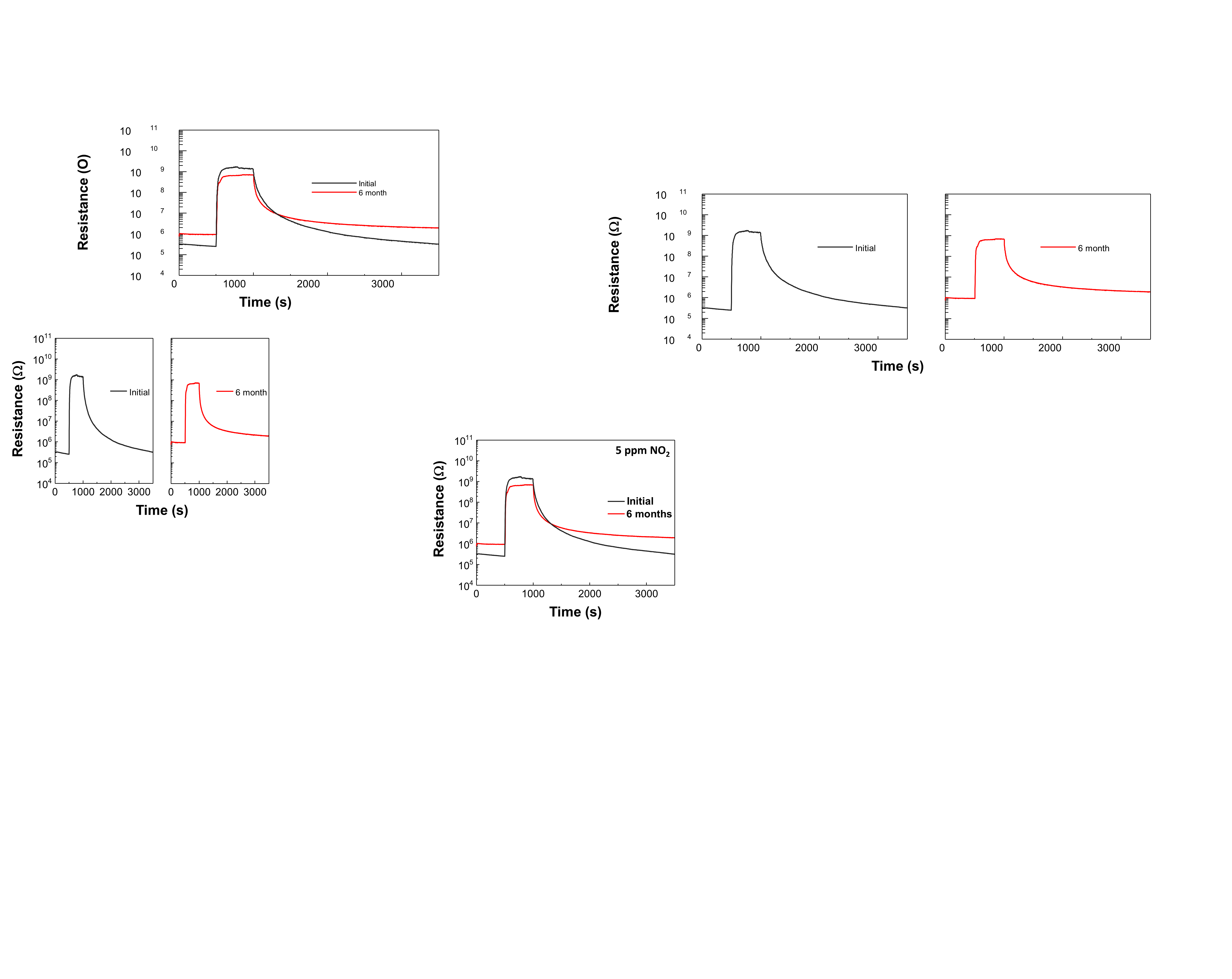


**Fig. S19** Long-term stability of SnO_2_ NPs in response to 5 ppm of NO_2_ at initial state and after 6 months

**Table S1** Summarized NO_2_ sensing performance of light-activated gas sensors

| **Material** | **Power consumption**  **(mW)** | **Light color (wavelength)** | **Response (concentration)** | **Response time/ recovery time** | **Refs.** |
| --- | --- | --- | --- | --- | --- |
| Ag/In_2_O_3_ | 0.000063 | Blue (435 nm) | 13.19 (1 ppm) | 54 min. / NA | [49] |
| ZnO | 0.184 | UV (390 nm) | 6.05 (2 ppm) | 75 min. / NA | [50] |
| TiO_2_ | 82.5 | UV (405 nm) | 30.58 (5 ppm) | 428 s / NA | [51] |
| S-SnO_2_ | 216 | Blue (450 nm) | 418 (5 ppm) | 170 s / 64 s | [14] |
| ZnO/In_2_O_3_ | 690 | UV (365 nm) | 2.21 (5 ppm) | 100 s / NA | [52] |
| ZnO  In_2_O_3_  WO_3_ | 700 | UV (365 nm) | 0.4 (5 ppm)  0.7 (5 ppm)  16 (5 ppm) | 197 s / 300 s  300 s / 500 s  20 min. / 30 min. | [53] |
| Ag/ZnO | 3000 | Blue (470 nm) | 2.4 (1 ppm) | 230 s / NA | [54] |
| SnO_2_ | 0.063 | Blue (453 nm) | 6928 (5 ppm) | 47 s / 49 s | **This work** |


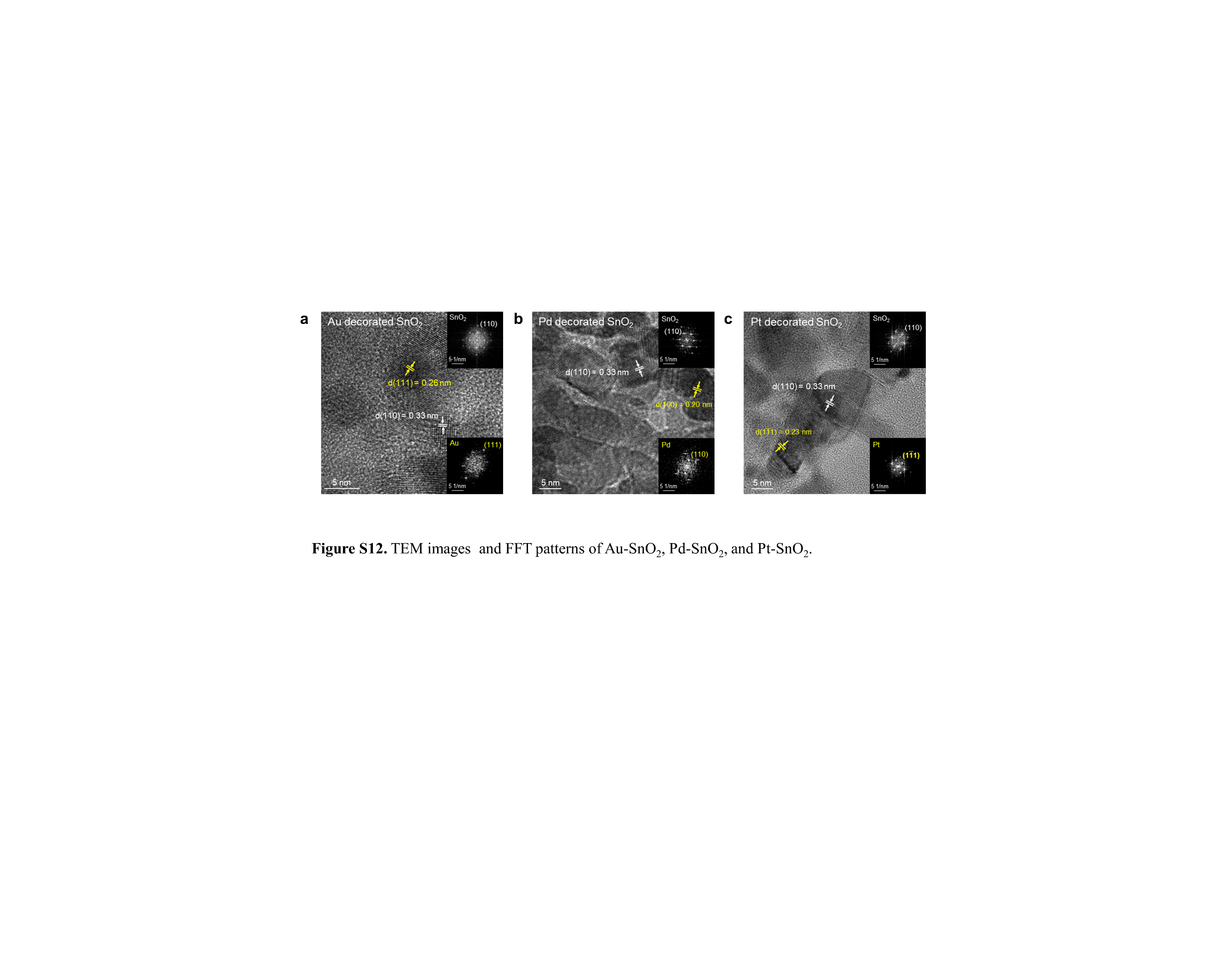
 **Fig. S20** TEM images and FFT patterns of **a** Au-SnO_2_ NPs, **b** Pd-SnO_2_ NPs, and **c** Pt-SnO_2_ NPs


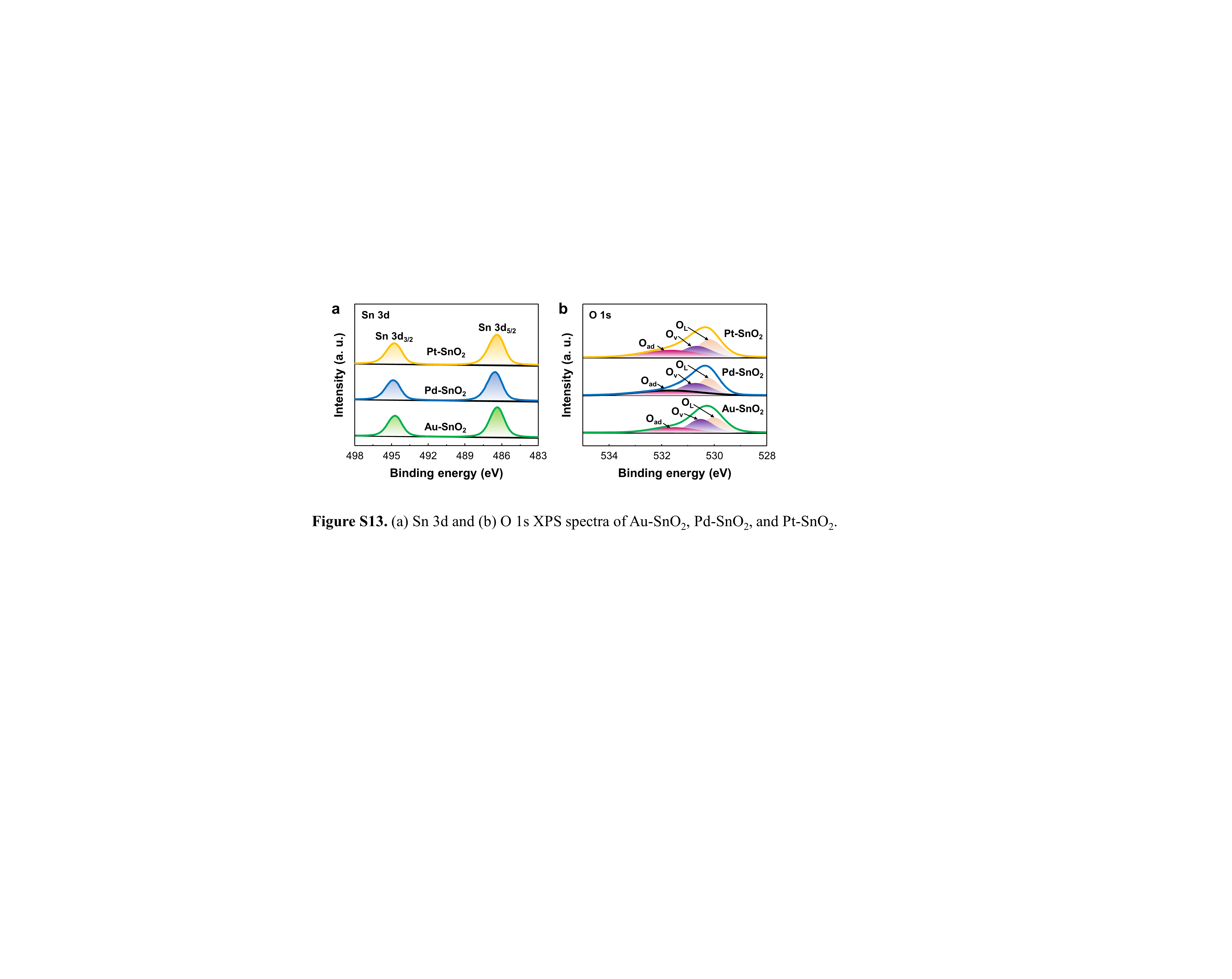


**Fig. S21** **a** Sn 3d and **b** O 1s XPS spectra of Au-SnO_2_ NPs, Pd-SnO_2_ NPs, and Pt-SnO_2_ NPs

**Table S2** Summarized table of the peak positions (eV) of Sn 3d and O 1s XPS spectra

| Element peak  Material | Sn 3d | | O 1s | | |
| --- | --- | --- | --- | --- | --- |
|  | Sn 3d_5/2_ | Sn 3d_3/2_ | O_L_ | O_V_ | O_ab_ |
| SnO_2_ | 486.65 | 495.05 | 530.25 | 530.75 | 531.75 |
| Au-SnO_2_ | 486.38 | 494.78 | 530.00 | 530.50 | 531.50 |
| Pd-SnO_2_ | 486.40 | 494.90 | 530.20 | 530.70 | 531.70 |
| Pt-SnO_2_ | 486.35 | 494.75 | 530.10 | 530.60 | 531.60 |

**Table S3** Summarized table of the peak positions of Au 4f, Pd 3d, and Pt 4f XPS spectra

| Photoelectron peak | Metallic state | Binding energy (eV) | Photoelectron peak | Metallic state | Binding energy (eV) | Photoelectron peak | Metallic state | Binding energy (eV) |
| --- | --- | --- | --- | --- | --- | --- | --- | --- |
| Au 4f_7/2_ | Au^0^ | 83.83 | Pd 3d_5/2_ | Pd^0^ | 335.43 | Pt 4f_7/2_ | Pt^0^ | 71.1 |
| Au 4f_5/2_ | Au^0^ | 87.5 | Pd 3d_3/2_ | Pd^0^ | 340.69 | Pt 4f_5/2_ | Pt^0^ | 74.43 |
| Au 4f_7/2_ | Au^3+^ | 86.8 | Pd 3d_5/2_ | Pd^2+^ | 336.48 | Pt 4f_7/2_ | Pt^2+^ | 72.2 |
| Au 4f_5/2_ | Au^3+^ | 90.47 | Pd 3d_3/2_ | Pd^2+^ | 341.74 | Pt 4f_5/2_ | Pt^2+^ | 75.53 |
|  |  |  | Pd 3d_5/2_ | Pd^4+^ | 337.43 |  |  |  |
|  |  |  | Pd 3d_3/2_ | Pd^4+^ | 342.69 |  |  |  |

_
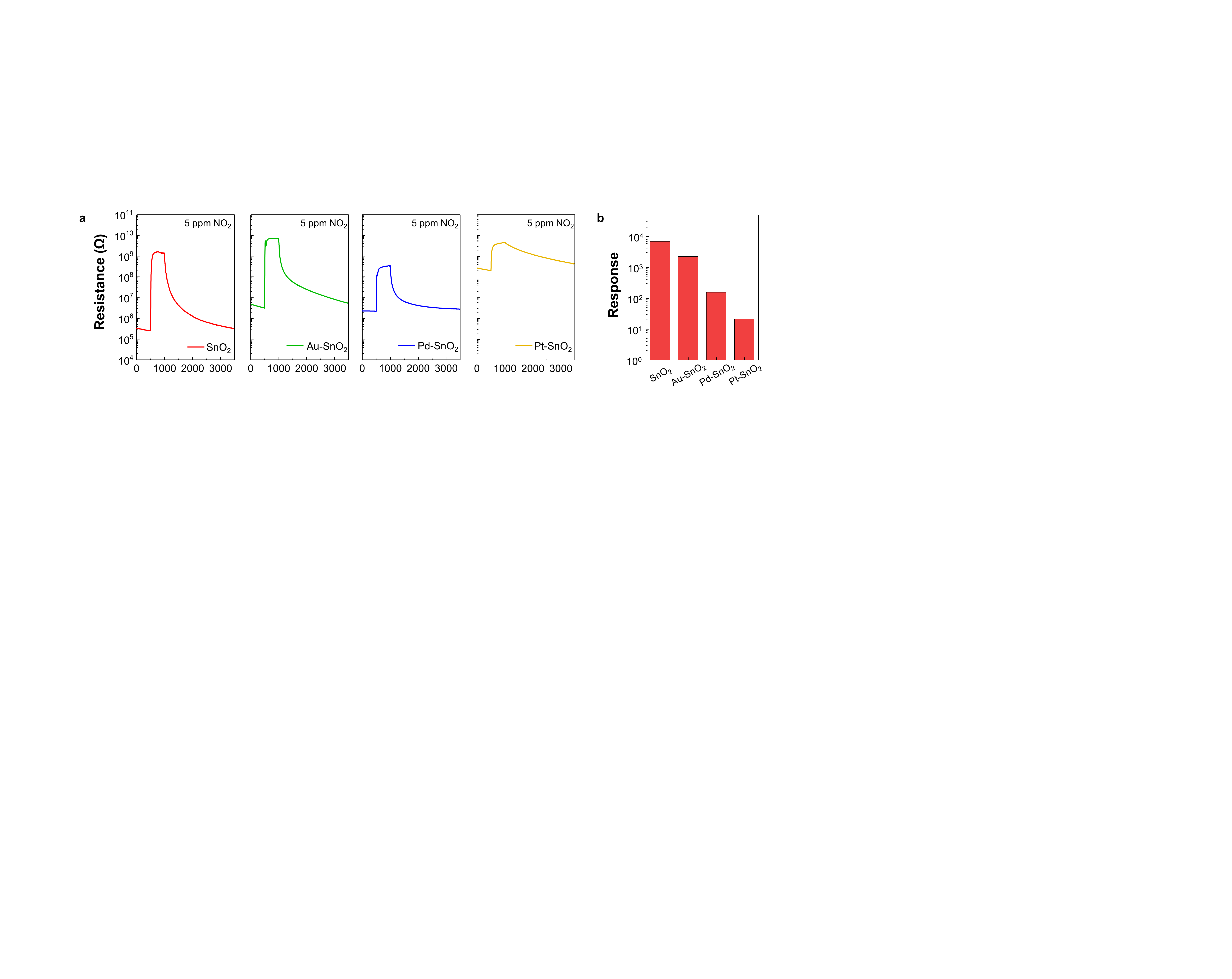
_**Fig. S22** **a** Dynamic curves and **b** response plots of SnO_2_ NPs, Au-SnO_2_ NPs, Pd-SnO_2_ NPs, and Pt-SnO_2_ NPs to 5 ppm of NO_2_


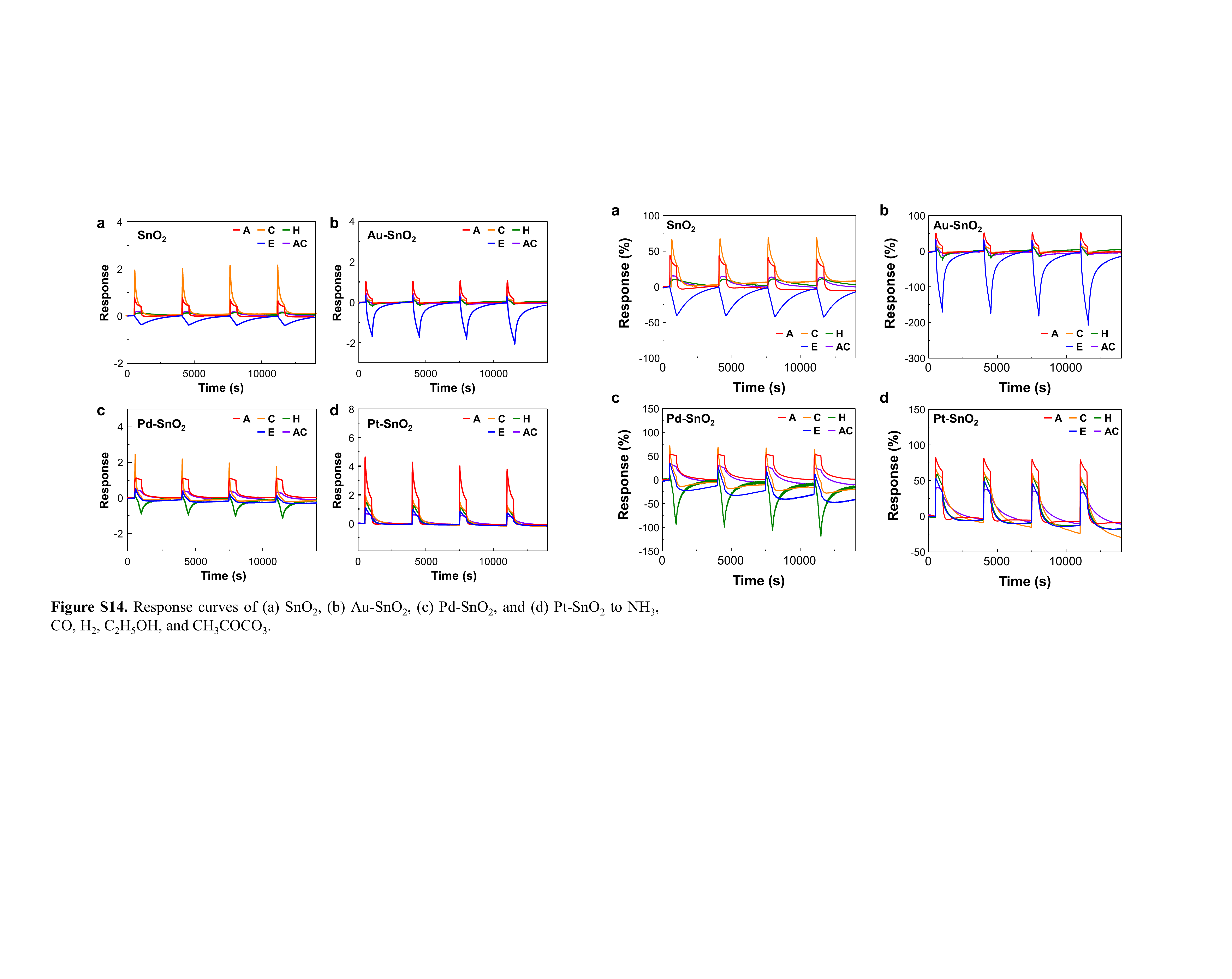


**Fig. S23** Response curves of **a** SnO_2_, **b** Au-SnO_2_ NPs, **c** Pd-SnO_2_ NPs, and **d** Pt-SnO_2_ NPs to 50 ppm of NH_3_, CO, H_2_, C_2_H_5_OH, and CH_3_COCH_3_


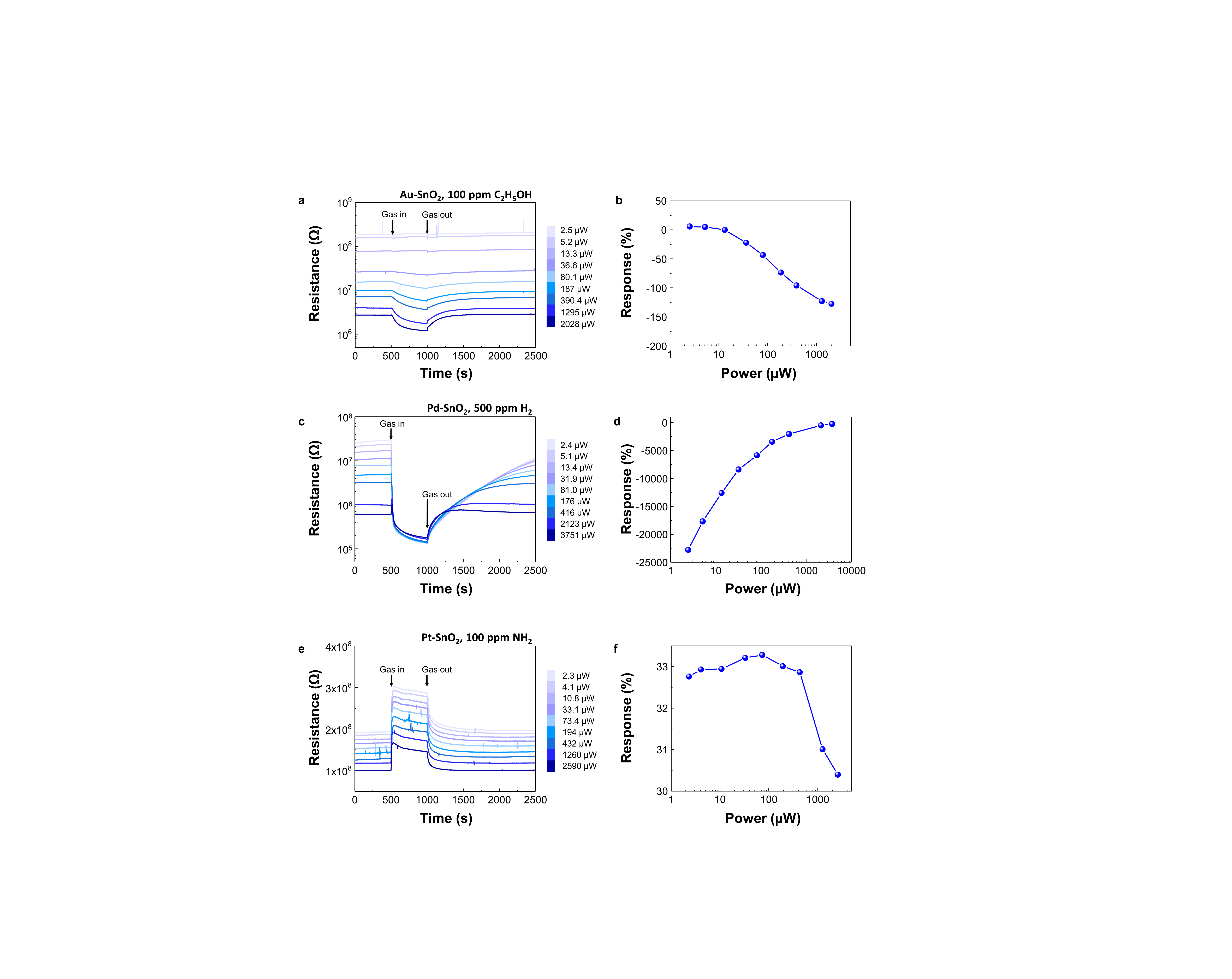


**Fig. S24** The gas sensing properties of gas sensor array based on noble metal decoration under various light intensities. **a** The dynamic curves and **b** response plot of Au-SnO_2_ NPs to 100 ppm of C_2_H_5_OH. **c** The dynamic curves and **d** response plot of Pd-SnO_2_ NPs to 500 ppm of H_2_. **e** The dynamic curves and **f** response plot of Pt-SnO_2_ NPs to 100 ppm of NH_3_

The sensing properties of the gas sensor array based on noble metal decoration were measured under various intensities. Au-SnO_2_ NPs was measured with 100 ppm of C_2_H_5_OH, Pd-SnO_2_ NPs was measured with 500 ppm of H_2_, and Pt-SnO_2_ NPs was measured with 100 ppm of NH_3_. For Au-SnO_2_ NPs and Pd-SnO_2_ NPs, as shown in Fig. S24a-d, the response plots to light intensity did not exhibit the volcano shape observed in Fig. 2b. The volcano shape of the response plot is related to the activation layer of SnO_2_ NPs by light illumination. As explained in Fig. 2c, when the excessive light intensity illuminates SnO_2_, most NO_2_ reacts at the top layer, which leads to a higher resistance in top layer than in bottom layer. Thus, the response decreased because current flows through bottom layer.

However, in the case of Au-SnO_2_ NPs with C_2_H_5_OH, and Pd-SnO_2_ NPs with H_2_, the resistance decreased with the input of gases. Therefore, the response tendency to light intensity is different from SnO_2_ NPs to NO_2_. For Au-SnO_2_ NPs with C_2_H_5_OH, the response increased as light intensity increased. The increase in light intensity enhanced the generation of photo-activated oxygen, which led to high reactivity with C_2_H_5_OH, as explained in Supplementary Note 1. Even the most C_2_H_5_OH reacted on top layer due to excessive light intensity, the response increased because the current flows through top layer, which has a lower resistance than bottom layer.

For Pd-SnO_2_ NPs with H_2_, the response decreased as the light intensity increased. This tendency is explained by the sensing mechanism of Pd-SnO_2_, which is related to the phase transition of Pd to PdH_x_ [S12]. Cai et al. interpreted the sensing mechanism of Pd-SnO_2_ NPs with H_2_ by changing band structure through phase transition of Pd to PdH_x_. As the work function of Pd (5.6 eV) [S13] is larger than the fermi level of SnO_2_ (4.15 eV) [S14], Schottky junction is formed with depletion layer between Pd and SnO_2_. When H_2_ is introduced to Pd-SnO_2_ NPs, Pd transforms into PdH_x_, changing the work function of Pd from 5.6 eV to 3.2 eV [S15]. Since the work function of PdH_x_ is lower than the fermi energy of SnO_2_, Schottky junction transforms into ohmic junction. Accordingly, the depletion layer between Pd and SnO_2_ disappears, leading to a decrease in the resistance of Pd-SnO_2_ NPs. When light illuminates Pd-SnO_2_ NPs, the photo-activated oxygen is generated by photocurrent and becomes reactive with H^+^ ion adsorbed in Pd, increasing desorption of H_2_ on Pd. This may affect the phase transition of Pd to PdH_x_, resulting in a decrease in response.

For Pt-SnO_2_ NPs with NH_3_, the response plots to light intensity showed volcano shape, as shown in Fig. S24e, f. The optimal power of µLED at highest response was 73.4 µW, which is close to the optimal power of SnO_2_ NPs with NO_2_. As the resistance of Pt-SnO_2_ NPs increased with the input of NH_3_, the mechanism is the same as the SnO_2_ NPs to NO_2_, as explained in Fig. 2c.

**
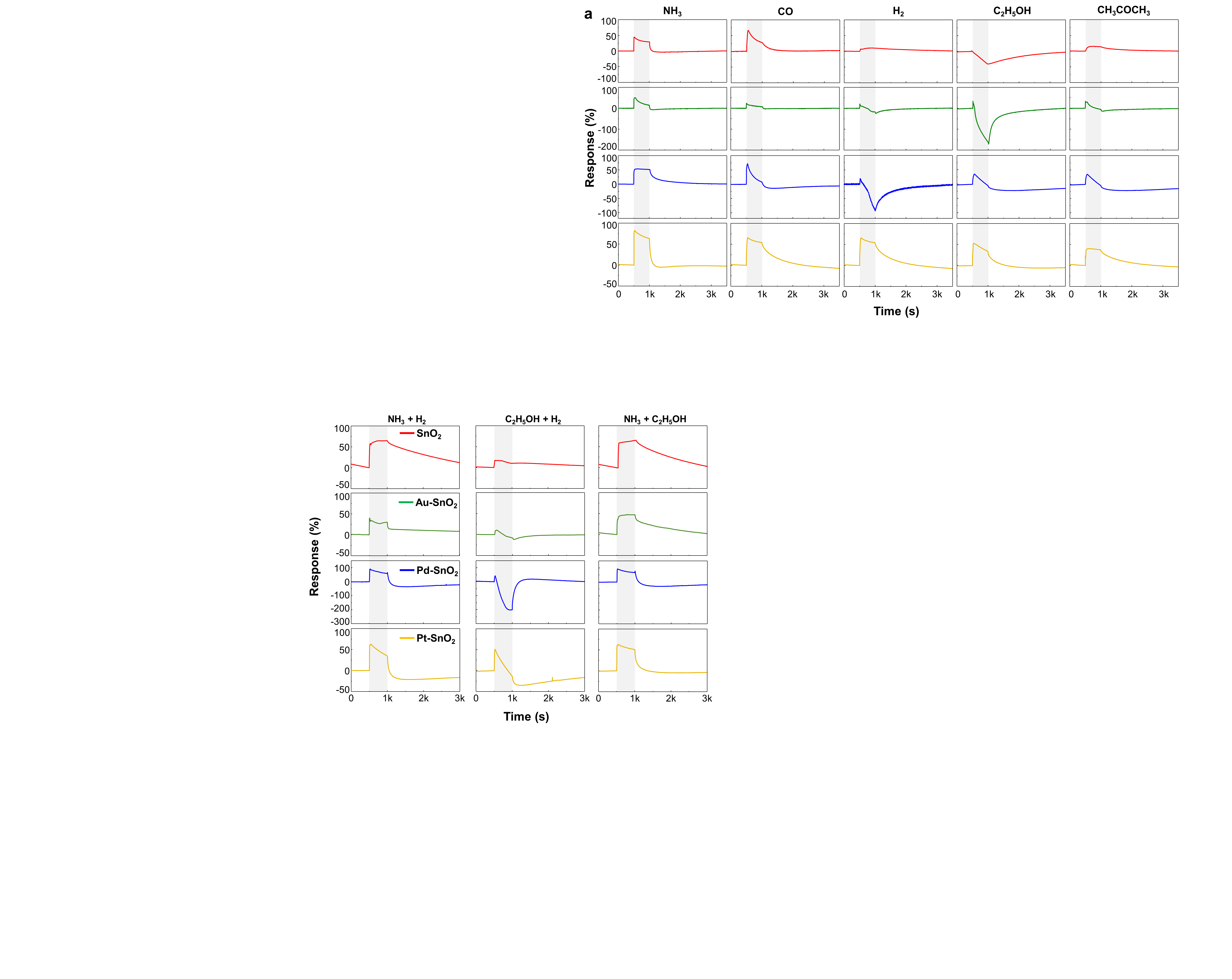
**

**Fig. S25** Response curves of SnO_2_ NPs, Au-SnO_2_ NPs, Pd-SnO_2_ NPs, and Pt-SnO_2_ NPs to gas mixtures containing 50 ppm of NH_3_, H_2_, and C_2_H_5_OH


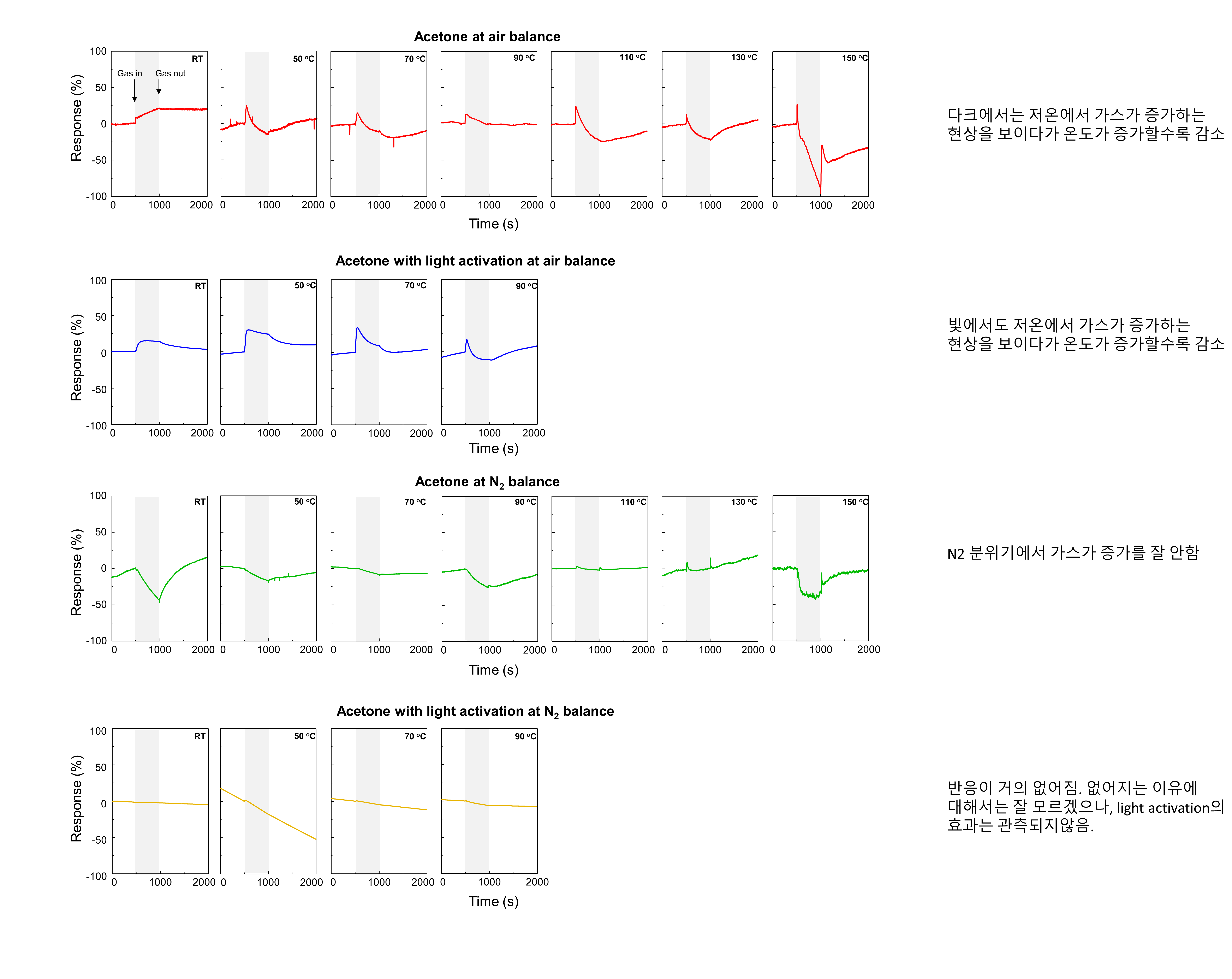


**Fig. S26** Response curves of SnO_2_ to 50 ppm of CH_3_COCH_3_ under various conditions of temperature, light, and atmosphere


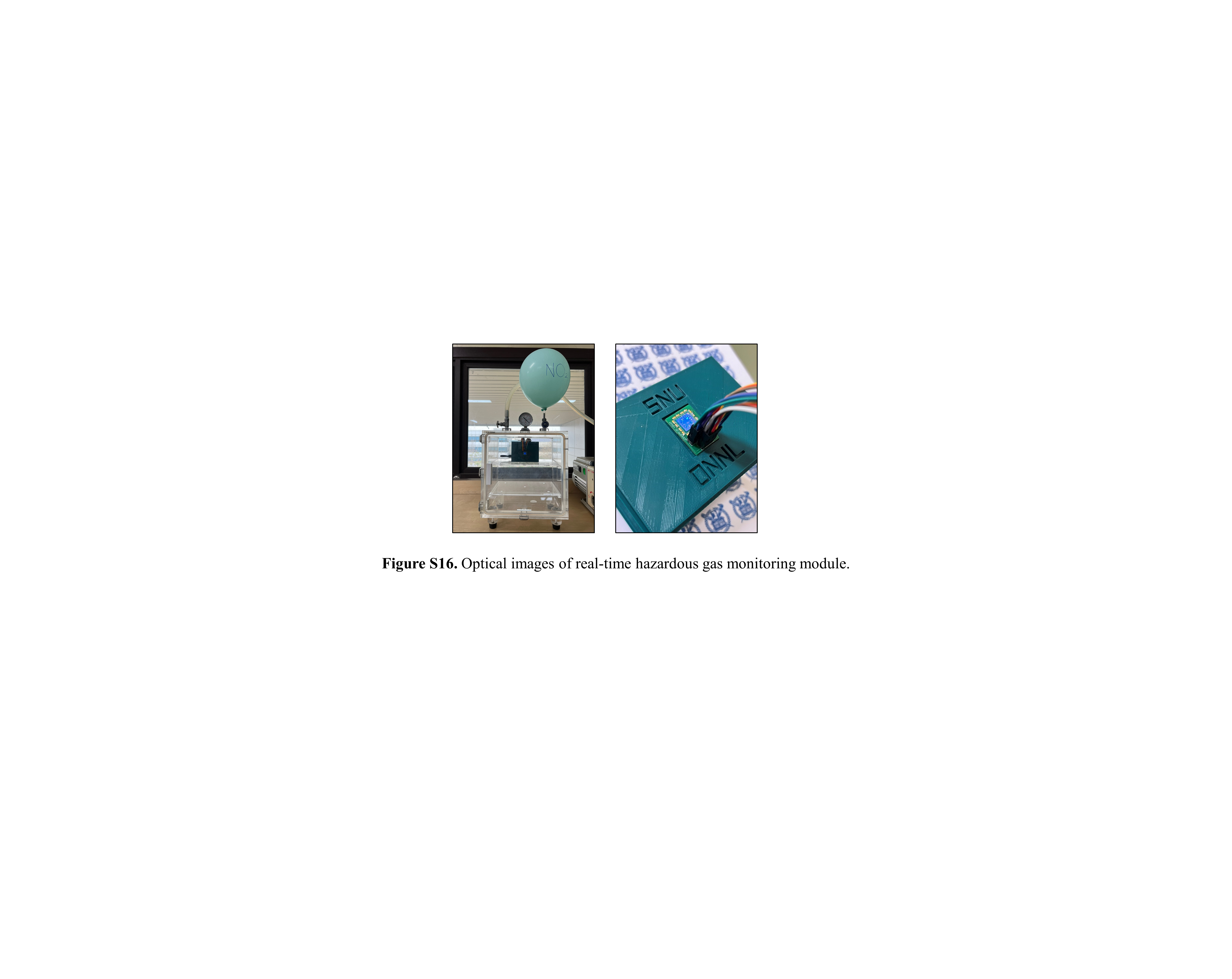


**Fig. S27** Optical images of real-time hazardous gas monitoring module


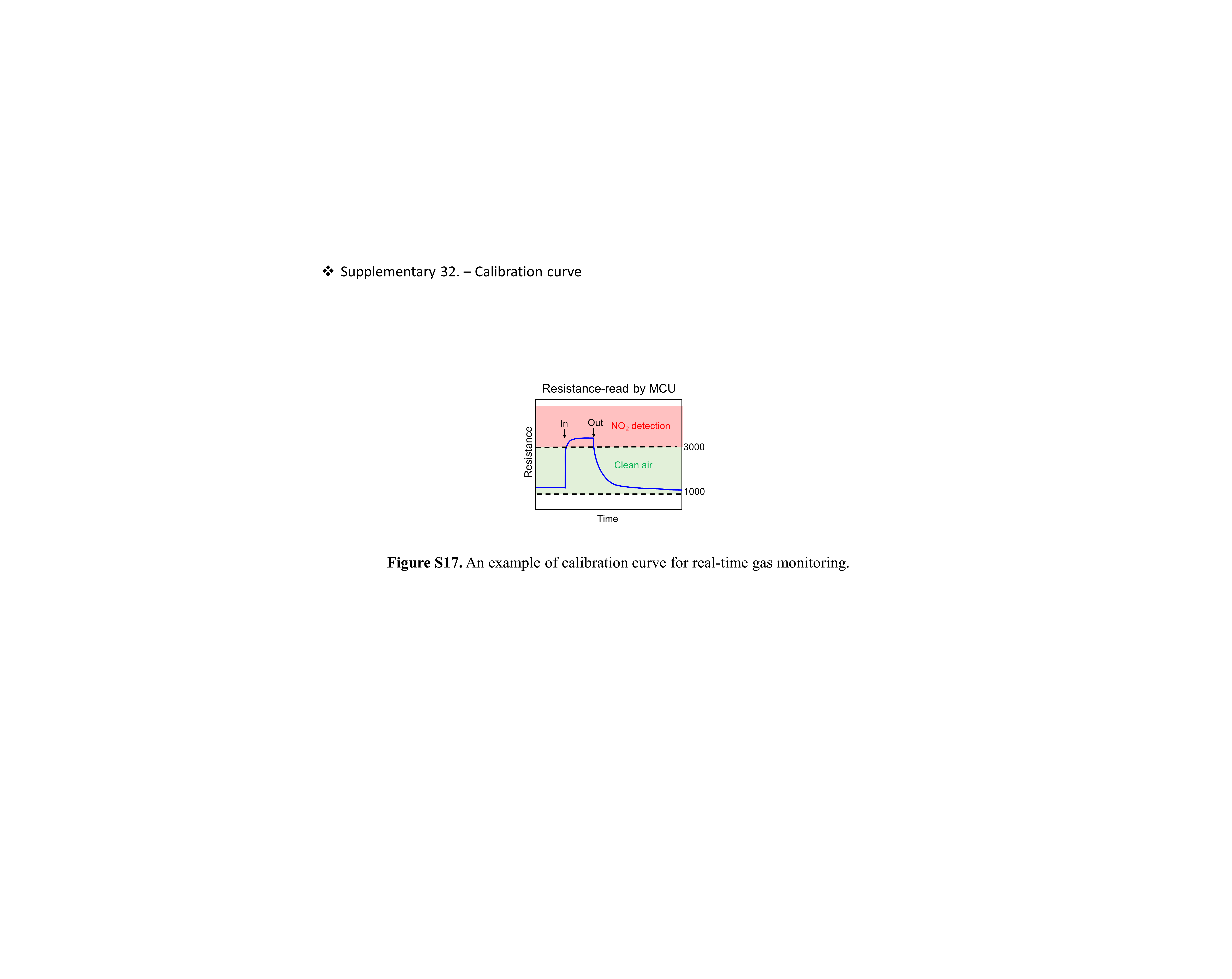


**Fig. S28** Example of calibration curve for real-time gas monitoring

**Note S3 Calibration of MCU for real-time gas monitoring**

Our integrated gas sensor array operates by resistance-read process on a real-time basis. The micro-controller unit (MCU) processor has a 12-bit analog-to-digital (ADC) to read the voltage signal compared to the reference resistor and normalized their resistance from 0 to 4095. Following the light-activated gas-sensing properties of our sensor array, the gas hazard threshold resistance can be estimated and preprogrammed as a normalized value in our MCU. During the real-time monitoring operation, our MCU can transmit the measured value to the mobile phone through the Wi-Fi. The red light turns on when NO_2_ is detected, the green light turns on when C_2_H_5_OH is detected, the blue light turns on when H_2_ is detected, and the yellow light turns on when NH_3_ is detected. The above MCU processing was programmed in C++.


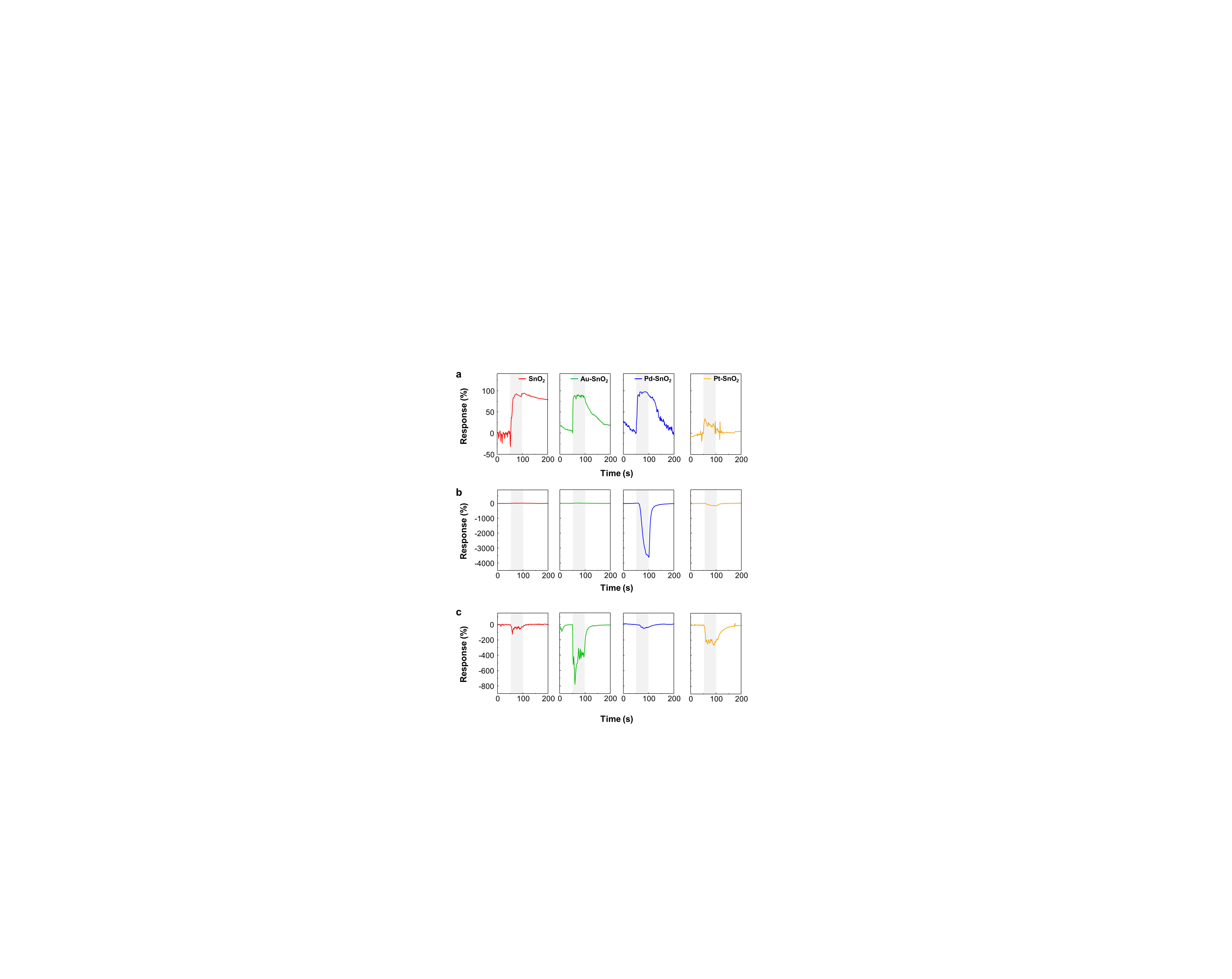


**Fig. S29** Response plots of practical application of a µLED gas sensor array to **a** ferment skate, **b** H_2_ leakage, and **c** wine

**Supplementary References**

1. S. Kim, J.-H. In, S. H. Kim, K. Han, D. Lim et al., Study of high transmittance of SiO_2_/Nb_2_O_5_ multilayer thin films deposited by plasma-assisted reactive magnetron sputtering. Appl. Sci. **13**(24), (2023). <https://doi.org/10.3390/app132413271>
2. N. Barsan, U. Weimar, Conduction model of metal oxide gas sensors. J. Electroceramics **7**, 143-167 (2001). [https://doi.org/10.1023/A:1014405811371](https://doi.org/https://doi.org/10.1023/A:1014405811371)
3. D. Gu, X. Wang, W. Liu, X. Li, S. Lin et al., Visible-light activated room temperature NO_2_ sensing of SnS_2_ nanosheets based chemiresistive sensors. Sensors Actuators B: Chemical **305**, 127455 (2020). <https://doi.org/10.1016/j.snb.2019.127455>
4. A. Staerz, U. Weimar, N. Barsan, Current state of knowledge on the metal oxide based gas sensing mechanism. Sensors and Actuators B: Chemical **358**, 131531 (2022). <https://doi.org/10.1016/j.snb.2022.131531>
5. S. Park, M. Kim, Y. Lim, D. Oh, J. Ahn et al., Dual-photosensitizer synergy empowers ambient light photoactivation of indium oxide for high-performance NO_2_ sensing. Adv. Mater. **36**(24), e2313731 (2024). <https://doi.org/10.1002/adma.202313731>
6. R. Kumar, N. Goel, M. Kumar, UV-activated MoS_2_ based fast and reversible NO_2_ sensor at room temperature. ACS Sensors **2**(11), 1744-1752 (2017). [https://doi.org/10.1021/acssensors.7b00731](https://doi.org/https://doi.org/10.1021/acssensors.7b00731)
7. X. Tian, X. Yang, F. Yang, T. Qi, A visible-light activated gas sensor based on perylenediimide-sensitized SnO_2_ for NO_2_ detection at room temperature. Colloids and Surfaces A: Physicochemical and Engineering Aspects **578**, 123621 (2019). [https://doi.org/10.1016/j.colsurfa.2019.123621](https://doi.org/https://doi.org/10.1016/j.colsurfa.2019.123621)
8. S. Park, S. Jeon, H. Kim, J. Philips, D. Oh et al., Imparting metal oxides with high sensitivity toward light‐activated NO_2_ detection via tailored interfacial chemistry. Adv. Funct. Mater. **33**(17), 2214008 (2023). <https://doi.org/10.1002/adfm.202214008>
9. Y. Zhong, W. Li, X. Zhao, X. Jiang, S. Lin et al., High-response room-temperature NO_2_ sensor and ultrafast humidity sensor based on SnO_2_ with rich oxygen vacancy. ACS Appl. Mater. Interfaces **11**(14), 13441-13449 (2019). <https://doi.org/10.1021/acsami.9b01737>
10. C. Xu, J. Tamaki, N. Miura, N. Yamazoe, Grain size effects on gas sensitivity of porous SnO_2_-based elements. Sensors Actuators B: Chemical **3**(2), 147-155 (1991). [https://doi.org/10.1016/0925-4005(91)80207-Z](https://doi.org/https://doi.org/10.1016/0925-4005(91)80207-Z)
11. A. Rothschild, Y. Komem, The effect of grain size on the sensitivity of nanocrystalline metal-oxide gas sensors. Journal of Appl. Phys. **95**(11), 6374-6380 (2004). [https://doi.org/10.1063/1.1728314](https://doi.org/https://doi.org/10.1063/1.1728314)
12. S. H. Cho, J. M. Suh, B. Jeong, T. H. Lee, K. S. Choi et al., Substantially accelerated response and recovery in Pd-decorated WO_3_ nanorods gasochromic hydrogen sensor. Small e2309744 (2024). <https://doi.org/10.1002/smll.202309744>
13. J. Baek, B. Jang, M. H. Kim, W. Kim, J. Kim et al., High-performance hydrogen sensing properties and sensing mechanism in Pd-coated p-type Si nanowire arrays. Sensors Actuators B: Chemical **256**, 465-471 (2018). <https://doi.org/10.1016/j.snb.2017.10.109>
14. T. H. Eom, S. H. Cho, J. M. Suh, T. Kim, J. W. Yang et al., Visible light driven ultrasensitive and selective NO_2_ detection in tin oxide nanoparticles with sulfur doping assisted by l‐cysteine. Small **18**(12), 2106613 (2022). https://doi.org/10.1002/smll.202106613
15. X. Tang, P. A. Haddad, N. Mager, X. Geng, N. Reckinger et al., Chemically deposited palladium nanoparticles on graphene for hydrogen sensor applications. Sci. Rep. **9**(1), 3653 (2019). <https://doi.org/10.1038/s41598-019-40257-7>
